# Roles of microbiota in autoimmunity in *Arabidopsis* leaves

**DOI:** 10.1038/s41477-024-01779-9

**Published:** 2024-09-06

**Authors:** Yu Ti Cheng, Caitlin A. Thireault, Li Zhang, Bradley C. Paasch, Reza Sohrabi, Sheng Yang He

**Affiliations:** 1 Department of Biology, Duke University, Durham, NC USA; 2 Howard Hughes Medical Institute, Duke University, Durham, NC USA; 3 Department of Energy Plant Research Laboratory, Michigan State University, East Lansing, MI USA; 4 School of Biology and Basic Medical Science, Soochow University, Suzhou, China

**Keywords:** Biotic, Microbiology

## Abstract

Over the past three decades, researchers have isolated plant mutants that show constitutively activated defence responses in the absence of pathogen infection. These mutants are called autoimmune mutants and are typically dwarf and/or bearing chlorotic/necrotic lesions. Here, from a genetic screen for *Arabidopsis* genes involved in maintaining a normal leaf microbiota, we identified *TIP GROWTH DEFECTIVE 1* (*TIP1*), which encodes an S-acyltransferase, as a key player in guarding leaves against abnormal microbiota level and composition under high-humidity conditions. The *tip1* mutant has several characteristic phenotypes of classical autoimmune mutants, including a dwarf stature, showing lesions, and having a high basal level of defence gene expression. Gnotobiotic experiments revealed that the autoimmune phenotypes of the *tip1* mutant are largely dependent on the presence of microbiota as axenic *tip1* plants have markedly reduced autoimmune phenotypes. We found that the microbiota dependency of autoimmune phenotypes is shared by several ‘lesion mimic’-type autoimmune mutants in *Arabidopsis*. It is worth noting that autoimmune phenotypes caused by mutations in two *Nucleotide-Binding*, *Leucine-Rich Repeat* (*NLR*) genes do not require the presence of microbiota and can even be partially alleviated by microbiota. Our results therefore suggest the existence of at least two classes of autoimmunity (microbiota-dependent versus microbiota-independent) in plants. The observed interplay between autoimmunity and microbiota in the lesion mimic class of autoimmunity is reminiscent of the interactions between autoimmunity and dysbiosis in the animal kingdom. These parallels highlight the intricate relationship between host immunity and microbial communities across various biological systems.

## Abstract (web summary)

The authors show that dysbiotic microbiota can cause severe autoimmunity conditions in *Arabidopsis* plants, a concept that is reminiscent of autoimmunity–dysbiosis interactions found in mammals.

## Main

In the past 40 years, tremendous progress has been made in the understanding of plant immune responses against pathogens^1–3^. The plant innate immune system comprises both constitutive physical barriers and inducible immune responses. Inducible immunity can be initiated by plasma membrane-residing receptor kinases that recognize conserved microbe-associated molecular patterns, resulting in a broad-spectrum basal immunity called pattern-triggered immunity (PTI). Successful pathogens have evolved virulence-associated effector molecules to defeat the plant immune system and/or to create a conducive microenvironment within the host tissue as two major pathogenic mechanisms^4–7^. In response, plants have evolved an array of intracellular nucleotide-binding leucine-rich repeat (NLR) immune receptors that recognize the presence of specific pathogen effectors, leading to effector-triggered immunity that often involves hypersensitive cell death^2,8^. Activation and mutual potentiation of PTI and effector-triggered immunity lead to accumulation of defence hormones, including salicylic acid, activation of defence gene expression and other cellular responses^8,9^. Activation of plant immune response is often accompanied by growth inhibition, a phenomenon known as growth–defence trade-offs^10,11^.

In nature, plants spend most of their life in an environment that is occupied by enormously diverse, mostly non-pathogenic (commensal) microorganisms^12,13^. Plant-associated commensal microbial community plays a vital role in influencing host growth, development and stress responses^14,15^. However, compared with plant–pathogenic microbe interactions, less is known about how plants recognize and communicate with their surrounding non-pathogenic microbial communities and how plants fine tune their immune system to achieve a long-term, harmonious state in the context of complex microbial communities. Only in the past decade, thanks to the advent of high-throughput sequencing technologies and availability of genetic mutants in model systems, such as *Arabidopsis* and rice, increasing efforts are being devoted to the study of the interplay between plant host genetics and associated microbial communities^12,14,16^. Nevertheless, the mechanisms of plant–microbiome interactions in terms of (1) how plants recognize, configure and maintain a homeostatic composition of their associated microbiota and (2) how the host immune system distinguishes non-self signals derived from commensal versus pathogenic microbes are still largely unclear.

The lifestyle of commensal bacterial microbiota in plant leaves resembles those of non-pathogenic strains of phyllosphere bacterial phytopathogens: both are adapted to live in plant tissues but are unable to multiply to a high population level in planta. Recent studies have shown that the transcriptomes of commensal bacteria in planta share a high degree of similarity to that of a non-pathogenic mutant of the phyllosphere-adapted bacterial pathogen *Pseudomonas syringae* pv. *tomato* (*Pst*) DC3000^17,18^. Furthermore, two *Arabidopsis* quadruple mutants, *min7 fls2 efr cerk1* (*mfec*) and *min7 bak1 bkk1 cerk1* (*mbbc*), which are defective in PTI and MIN7-associated intracellular vesicle trafficking, not only fail to control the proliferation of non-pathogenic mutants of *Pst* DC3000 but also are unable to maintain a typical endophytic leaf microbiota^19,20^. In addition, immunity-associated reactive oxygen species (ROS) is required for maintaining a homeostatic leaf microbiota as the *Arabidopsis rbohD* mutant, which is defective in the generation of immunity-associated ROS, has altered leaf microbiota composition^21^. Taken together, these initial studies begin to identify plant genes/pathways that are required for maintaining a normal bacterial microbiota in *Arabidopsis* leaves and provide a strong link between plant immune regulation and microbiota homeostasis^15^.

To identify additional plant genes involved in regulating plant–microbiome interactions and microbiota homeostasis, we conducted a forward genetic screen to isolate *Arabidopsis* mutants that show an altered response to non-pathogenic mutants of *Pst* DC3000 and endophytic leaf microbiota. Characterization of the resulting mutants led to an unexpected broad connection between microbiota and autoimmunity in plants.

## Results

### Identifying *Arabidopsis* mutants with altered leaf microbiota

We previously found that *Arabidopsis* mutant plants compromised in three pattern-recognition co-receptor genes, *BAK1*, *BKK1* and *CERK1*, are still capable of preventing over-proliferation of non-pathogenic strain *Pst* D28E^19^ and endogenous leaf microbiota^20^, suggesting that genes whose functions are either independent of and/or additive to these three pattern-recognition co-receptors are involved in plant interactions with non-pathogenic microbes. We therefore conducted a forward genetic screen in the *bak1-5 bkk1-1 cerk1-2* triple mutant background^22,23^ (*bbc* hereafter), intending to identify such genes (see Supplementary Fig. 1 for the setup of the genetic screen).

**Extended Data Fig. 1:**
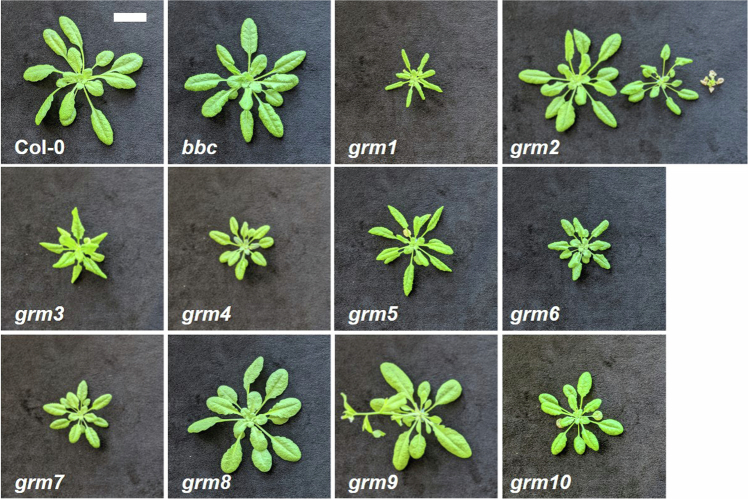
Mutants isolated from the *bbc* genetic screen. Plant images of 4-week-old soil grown *grm* mutants; scale bar indicates 2 cm. All *grm* mutant phenotypes were inherited as recessive traits with exception of *grm2*. *grm2* phenotypes were inherited as semi-dominant trait; based on the 1:2:1 segregation ratio, the *grm2* mutant image panel contains *bbc*-like (left), heterozygous (middle), and homozygous (right) plants.

We carried out the primary screen by flood-inoculating 3-week-old plate-grown M2 seedlings with *Pst* D28E^24^, a non-pathogenic mutant strain which was constructed by deleting 28 effector genes of *Pst* DC3000. Individual plants that showed disease-like symptoms (for example, necrosis or chlorosis) were transplanted to soil and grown for seeds. Secondary screen of putative mutants was conducted by monitoring disease-like symptoms after syringe infiltration of non-pathogenic *Pst* Δ*hrcC* mutant strain^25^ (*Pst* DC3000 defective in type III secretion of all effectors, including the 28 effectors absent in the *Pst* D28E mutant strain) into leaves of 4-week-old soil-grown M3 plants. Next, we monitored disease-like symptoms induced by natural soil-derived microbiota under holoxenic conditions in a peat-based gnotobiotic system^26^. In total, we identified ten mutants with various degrees of enhanced disease-like symptoms in response to non-pathogenic *Pst* DC3000 strains and/or natural soil-derived microbiota; we named them *guardian of normal microbiota* (*grm*) mutants (see Extended Data Fig. 1 for the morphology of soil-grown *grm* mutants).

### Characterization of the *grm1* mutant

**Fig. 1:**
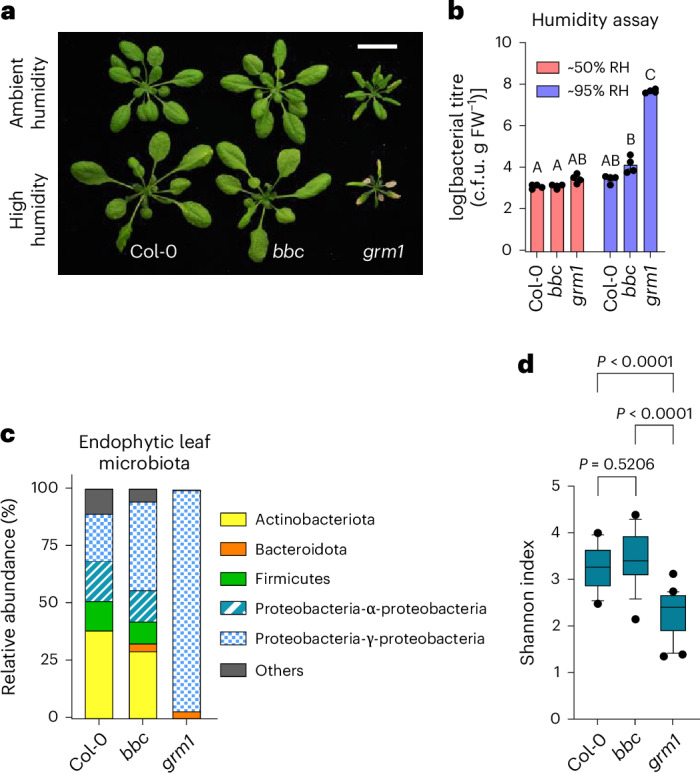
The appearance and leaf microbiota phenotypes of the *grm1* mutant. **a**, Top panel: 4-week-old soil-grown Col-0, *bbc* and *grm1* plants under ambient humidity (~50% RH) for 5 days (basal condition control). Bottom panel: 4-week-old soil-grown plants shifted to high humidity (~95% RH) for 5 days. Images were taken on day 5 of the humidity treatments. Scale bar, 2 cm. **b**, Population sizes of endophytic leaf microbiota after 5 days of indicated humidity conditions. Ambient humidity (~50% RH; basal condition control) and high humidity (~95% RH). Each column represents bacterial titres as log-transformed colony-forming units per gram of fresh weight (c.f.u. gFW^−1^). Data are displayed as mean ± s.e.m. (*n* = 4 biological replicates; each biological replicate contains 1–3 leaves from one plant). Different letters represent significant differences (*P* < 0.05, two-way analysis of variance (ANOVA) with Tukey’s honestly significant difference (HSD) test). Exact *P* values for all comparisons are shown in the Source data. Experiment was independently performed twice with similar results. **c**, Relative abundance of endophytic leaf bacteria at the phylum level and at the class level for Proteobacteria. **d**, Shannon indexes of endophytic leaf bacteria based on 16S rRNA gene amplicon sequence profiling of indicated genotypes. *n* = 11 (Col-0), *n* = 15 (*bbc*) and *n* = 20 (*grm1*) biological replicates for analysis of leaf endophytic bacterial microbiota. The centre lines of the box plot represent means, the box edges are the 75th and 25th percentiles, whiskers extend to 10–90 percentiles, and dots are outliers; statistical analysis was performed using one-way ANOVA with Tukey’s HSD test. Source data

Next, we conducted detailed characterization of one of the identified *grm* mutants, *grm1*. When grown in soil, the *grm1* mutant had a smaller stature compared with its progenitor, the *bbc* triple mutant. Notably, lower leaves that were in contact or in close proximity to the soil showed mild chlorosis (Fig. 1a). Previously, we found that *mfec* and *mbbc* mutant plants showed dysbiotic endophytic leaf microbiota and leaf tissue damage when grown under high humidity, a common environmental condition associated with plant disease outbreaks in nature^19,20^. The chlorotic lower leaves of the *grm1* mutant resembled those of *mfec* and *mbbc* mutant plants and prompted us to investigate whether *grm1* also shows dysbiotic endophytic leaf microbiota under high humidity. As expected in wild-type *Arabidopsis* (accession Col-0, which is the progenitor of the *bbc* triple mutant), characteristic hyponastic changes in leaf morphology were induced after 5 days of high-humidity treatment (~95% relative humidity (RH); Fig. 1a, bottom panel); however, high humidity did not cause over-proliferation of endophytic leaf microbiota, and no leaf chlorosis nor necrosis was observed (Fig. 1b). Like Col-0 plants, the *bbc* mutant also maintained a low level of culturable endophytic leaf microbiota, similar to these plants under ambient humidity (~50% RH). By contrast, after 5 days of high-humidity treatment, most of the *grm1* true leaves showed strong chlorosis (Fig. 1a, bottom panel). Quantification of culturable endophytic microbiota loads by colony counts on R2A agar plates revealed that, compared with Col-0 and the *bbc* mutant, the endophytic microbiota within leaves of *grm1* plants grown under high humidity was more than three orders of magnitude higher (Fig. 1b). In addition to a drastic increase in leaf endophytic microbiota, the relative abundance of leaf microbiota members in the *grm1* mutant also shifted overwhelmingly to Proteobacteria (Fig. 1c, ~97% in *grm1* leaves compared with ~45–55% in Col-0 and *bbc* leaves). In the *grm1* mutant, amplicon sequence variants (ASVs) in Proteobacteria belong predominantly to the genus *Pseudomonas* of the class Gammaproteobacteria, while *Bacillus* and *Paenibacillus* belonging to Firmicutes became nearly undetectable (Fig. 1c and Supplementary Data 1). Shannon index that measures richness and evenness of a microbial community composition also decreased in the *grm1* mutant, reflecting the overwhelming presence of Proteobacteria (Fig. 1d).

### Identification of the causal mutation in the *grm1* mutant

**Fig. 2:**
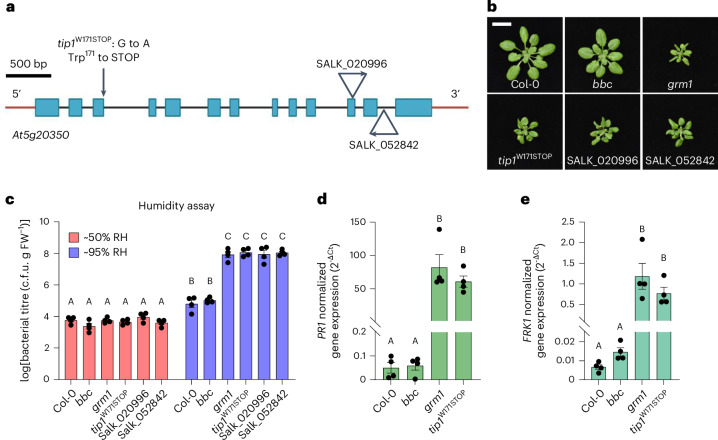
Characterization of *tip1* single mutant plants. **a**, A schematic diagram showing various mutant alleles in the *TIP1* gene. *tip1*^W171STOP^ is the allele isolated from this study which contains a G-to-A mutation at the splicing junction that is expected to cause a premature STOP codon at amino acid residue Trp^171^ in the ankyrin-repeat domain. SALK_020996 and SALK_052842 are T-DNA insertion alleles obtained from the *Arabidopsis* Biological Resource Center. **b**, Images of 4-week-old, soil-grown Col-0, *bbc*, *grm1* and *tip1* single mutant plants. Scale bar, 2 cm. **c**, Population sizes of endophytic leaf microbiota after 5 days under humidity conditions indicated. Ambient humidity (~50% RH; basal condition control) and high humidity (~95% RH). Results represent the mean values ± s.e.m. (*n* = 4 biological replicates; each biological replicate contains 1–3 leaves from one plant). Different letters represent a significant difference (*P* < 0.05, two-way ANOVA with Tukey’s HSD test). Exact *P* values for all comparisons are shown in the Source data. Experiment was independently performed twice with similar results. **d**,**e**, Normalized expression levels of immune-responsive *PR1* (**d**) and *FRK1* (**e**) genes in 4-week-old, soil-grown Col-0, *bbc*, *grm1* and *tip1* plants under ambient humidity (~50% RH). *PP2AA3* expression was used for normalization. *PR1* and *FRK1* normalized gene expression levels (2^−ΔCt^) were calculated by taking the difference between the cycle threshold (Ct) value of the *PR1* or *FRK1* gene and the Ct value of the internal control gene *PP2AA3*. This difference is used in the 2^−ΔCt^ formula to determine the relative expression levels of *PR1* and *FRK1*, normalized to the internal control. Results represent the mean values ± s.e.m. of four biological replicates. Each biological replicate is a pool of three plants. Different letters represent a significant difference (*P* < 0.05, one-way ANOVA with Tukey’s HSD test). Exact *P* values for all comparisons are shown in the Source data. Experiment was independently performed twice with similar results. Source data

**Extended Data Fig. 2:**
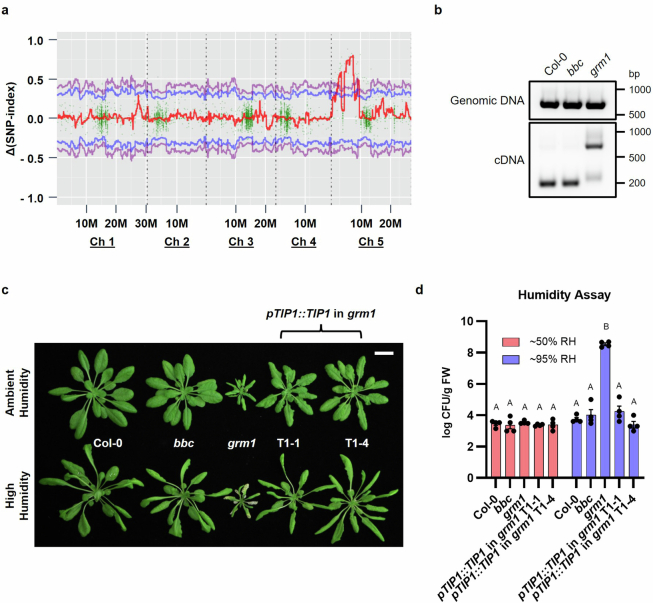
Identifying and confirming the causative mutation in the *grm1* mutant. **a**, *grm1* genomic mapping. Red line represents allele frequency. Blue and purple lines denote 95% and 99% confidence intervals, respectively. **b**, RT-PCR products using primers flanking the *grm1* mutation locus. Genomic (top panel) and complementary (bottom panel) DNA from indicated genotypes were used as templates. The retained intron is expected to lead to a premature STOP codon. **c**, Images of four-week-old, potting soil-grown Col-0, *bbc*, *grm1* and two independent *grm1* complementation lines under ambient humidity ( ~ 50% RH basal control condition; upper panel) or high humidity ( ~ 95% RH; bottom panel) for five days. Scale bar equals 2 cm. **d**, Transgene complementation of the *grm1* mutant. Population sizes of leaf endophytic microbiota after five days of plant growth under ambient humidity ( ~ 50% RH, basal control condition) or high humidity ( ~ 95% RH). Results represent the mean values ± SEM of four plants. Statistical analysis was performed using two-way ANOVA with Tukey’s HSD test. Exact p-values for all comparisons are shown in the Source Data. Experiment was independently performed twice with similar results. Source data Source data

**Extended Data Fig. 3:**
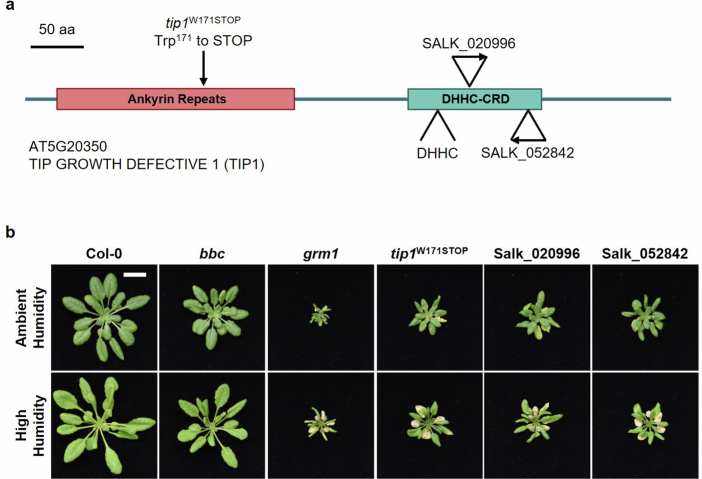
Appearance of *tip1* mutant alleles. **a**, A schematic diagram illustrating various mutant alleles of TIP1 protein. *tip1*^W171STOP^ is the allele isolated from this study which contains a G to A mutation at the splicing junction that is expected to cause a pre-mature STOP codon at amino acid residue Trp^171^ in the ankyrin-repeat domain. SALK_020996 and SALK_052842 have T-DNA insertions that would affect the catalytic DHHC cysteine-rich domain (DHHC-CRD). **b**, Images of four-week-old, potting soil-grown Col-0, *bbc*, *grm1* and various *tip1* single mutant plants under ambient humidity ( ~ 50% RH, basal control condition; upper panel) or high humidity ( ~ 95% RH) for five days. Scale bar equals 2 cm.

To identify the causative mutation in the *grm1* mutant, *grm1* plants were backcrossed with its progenitor, the *bbc* triple mutant, to generate a segregated F2 population. Analysing the mapping-by-sequencing data from *grm1* co-segregates revealed that the mutation was located on chromosome 5 between 5 Mb and 8 Mb with the allele frequency of the *grm1*-like pool peaking around 7 Mb (Extended Data Fig. 2a; see Supplementary Table 1 for a list of mutated loci in this region). Of all candidates, a G-to-A mutation on chromosome 5 at position 6,877,509 was tightly associated with the *grm1* phenotype and has the strongest effect. This G-to-A mutation occurs at the splicing junction of the 5′ end of the third intron of the *TIP GROWTH DEFECTIVE 1* (*TIP1*) gene and is expected to disrupt the splicing pattern, leading to a premature stop codon instead of tryptophan at position 171 (Fig. 2a and Extended Data Fig. 3a). PCR using *grm1* complementary DNA as template and primers flanking the mutation locus followed by Sanger sequencing revealed that, indeed, the mutation altered the splicing pattern of the *TIP1* transcript (Extended Data Fig. 2b). Transgene complementation with the full-length *TIP1* gene driven by its native promoter could complement *grm1* mutant phenotypes (that is, reversion of the dwarf stature and humidity-dependent dysbiotic phenotypes to those in *bbc* and wild-type Col-0), confirming the causative mutation in *grm1* is in the *TIP1* gene (Extended Data Figs. 2c,d).

To determine whether the dysbiotic phenotypes of the *grm1* mutant (that is, the *bbc tip1* quadruple mutant) are dependent on the background *bbc* mutations, we segregated the *tip1* mutation from the *bbc* triple mutations by outcrossing the *grm1* mutant with wild-type Col-0 plants and genotyping the resulting F2 population (see Supplementary Table 2 for primes used for genotyping). From The Arabidopsis Biological Resource Center^27^, we also obtained two independent *tip1* single mutant alleles (SALK_020996 and SALK_052842) carrying transfer DNA (T-DNA) insertions in the *TIP1* gene (Fig. 2a). All three *tip1* single mutants were larger than the original *grm1* (*bbc tip1*) mutant but still smaller than wild-type Col-0 plants (Fig. 2b). It is worth noting that the humidity-dependent dysbiosis phenotypes (for example, leaf chlorosis) (Extended Data Fig. 3b) and over-proliferation of endophytic leaf microbiota were still observed in all three alleles of *tip1* single mutant plants, suggesting dysbiotic phenotypes observed in *grm1* do not require *bbc* triple mutations in the background (Fig. 2c). As all three mutant alleles of *tip1* behaved similarly, we used *tip1*^W171STOP^ (*tip1* hereafter), the single mutant allele isolated from this screen, for all subsequent experiments.

### The *tip1* mutant has features of autoimmune mutants

**Extended Data Fig. 4:**
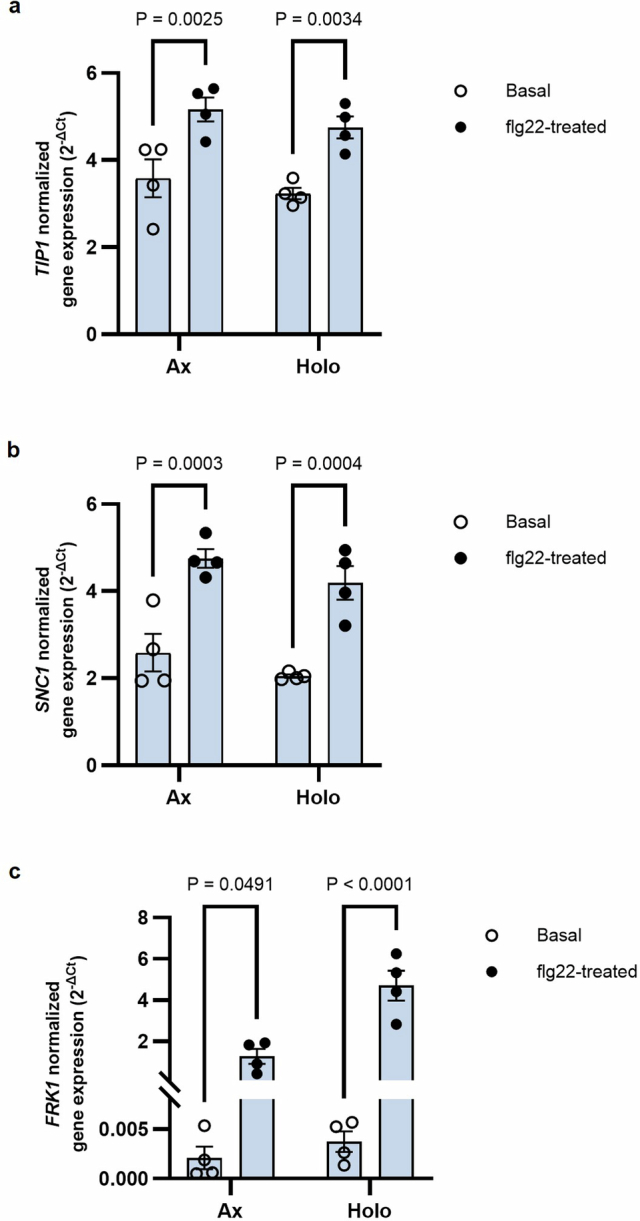
*TIP1* and *SNC1* gene expression is responsive to PTI elicitor flg22. Expression of *TIP1* (**a**), *SNC1* (**b**), and *FRK1* (**c**) in wild-type Col-0 plants after flg22 treatment. *PP2AA3* expression was used for normalization. Plants were grown under axenic (Ax) or holoxenic (Holo) conditions in GnotoPots. Open circles (∘) indicate the basal, uninduced state whereas closed circles (•) indicate the expression 90 min after 250 nM flg22 induction. Results represent the mean values ± SEM of four biological replicates. Statistical analysis was done by the two-way ANOVA with Fisher’s LSD test. Experiment was independently performed twice times with similar results. Source data

We noticed that the morphological phenotypes of *grm1* and *tip1* (that is, small statures and chlorotic leaves) were reminiscent of typical autoimmune mutants, which have been isolated in the past few decades. One hallmark of autoimmunity is constitutive high basal expression of immune-related marker genes in the absence of pathogen attacks. We therefore analysed two immune-related molecular markers, *Pathogenesis-Related Gene 1* (*PR1*) and *flg22-Induced Receptor-like Kinase 1* (*FRK1*). Indeed, both *grm1* and *tip1* plants have heightened *PR1* and *FRK1* expression under basal condition (that is, in the absence of pathogen inoculation) (Figs. 2d,e). It is worth noting that *TIP1* gene expression itself can be induced by flg22^28^, a flagellin-derived peptide that induces many PTI-associated genes^29,30^ (Extended Data Fig. 4). This suggests that *TIP1* is a flg22 responsive gene that is involved in maintaining leaf microbiota homeostasis.

### *tip1*- and *snc1*-mediated autoimmunity are distinct

We were intrigued by the morphological phenotypes and heightened immune-related marker gene expression of the *tip1* mutant as they point to a connection between dysbiosis and autoimmunity. However, it is not known (1) whether all autoimmune mutants possess a defect in maintaining microbiota homeostasis and/or (2) whether their autoimmune phenotype is dependent on microbiota. To investigate on a possible connection between altered microbiome and autoimmunity, we first examined microbiota-related phenotypes of a widely studied *Arabidopsis* autoimmune mutant, *snc1*, which contains a gain-of-function E552K mutation that results in an elevated level of the SNC1^E552K^ protein^31,32^.

**Fig. 3:**
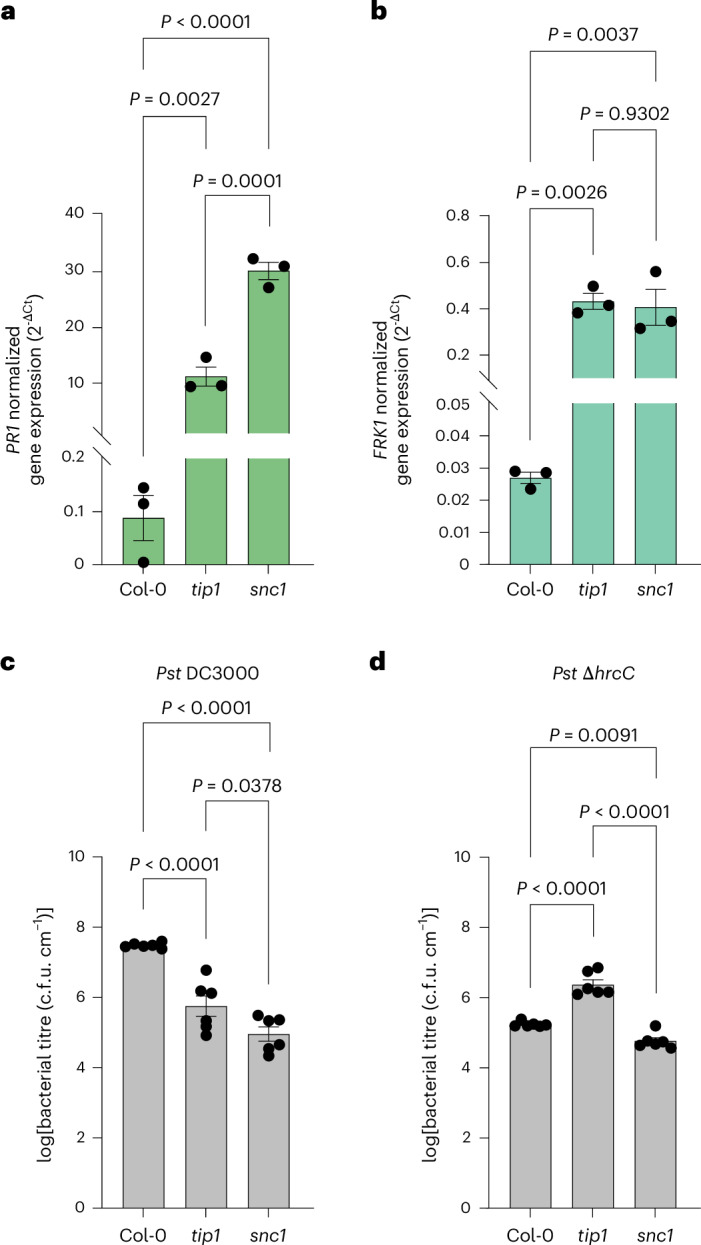
The autoimmune phenotypes of *tip1* and *snc1* mutants. **a**,**b**, Normalized expression levels of *PR1* (**a**) and *FRK1* (**b**) genes in 4-week-old, soil-grown Col-0, *tip1* and *snc1* plants under ambient humidity (~50% RH). *PP2AA3* was used for normalization. Results represent the mean values ± s.e.m. of three biological replicates. Each biological replicate is a pool of three plants. Statistical analysis was performed using one-way ANOVA with Tukey’s HSD test. Experiment was independently performed twice with similar results. **c**,**d**, Total bacterial populations in Col-0, *tip1* and *snc1* leaves 3 days after *Pst* DC3000 (**c**) or 5 days after *Pst* Δ*hrcC* (**d**) infiltration. Humidity was kept at ~95% throughout the duration of the disease assay. Each column represents bacterial titres as log-transformed colony-forming units per cm^2^ (c.f.u. cm^−^^2^) and is the mean of six biological replicates; each biological replicate contains leaf discs from infiltrated leaves from one plant; total of six plants were infiltrated. Error bars indicate s.e.m. Statistical analysis was performed using one-way ANOVA with Tukey’s HSD test. Experiment was independently performed twice with similar results. Source data

**Fig. 4:**
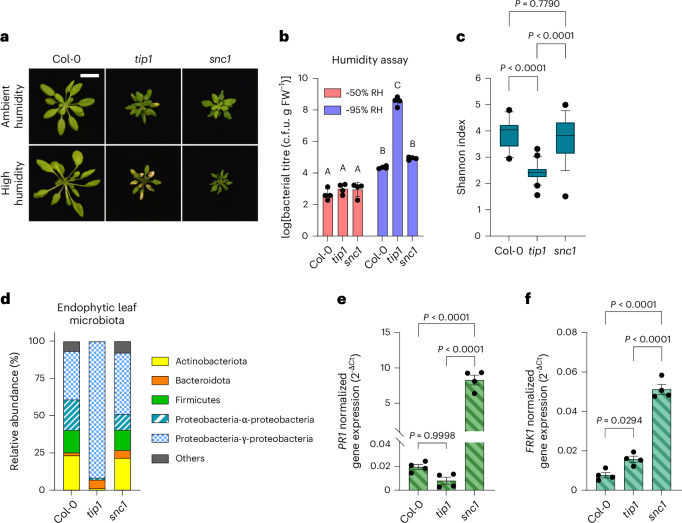
The distinct autoimmune phenotypes of *tip1* and *snc1* mutants. **a**, Top panel: 4-week-old, soil-grown Col-0, *tip1* and *snc1* plants under ambient humidity (~50% RH) for 5 days (basal condition control). Bottom panel: 4-week-old, soil-grown plants shifted to high humidity (~95% RH) for 5 days. Images were taken on day 5 of the treatments. Scale bar, 2 cm. **b**, Population sizes of endophytic leaf microbiota after 5 days of plant growth under humidity conditions indicated. Ambient humidity (~50% RH; basal condition control) and high humidity (~95% RH). Results represent the mean values ± s.e.m. of four biological replicates; each biological replicate contains one to two leaves from one plant. Different letters represent a significant difference (*P* < 0.05, two-way ANOVA with Tukey’s HSD test). Exact *P* values for all comparisons are shown in the Source data. Experiment was independently performed twice with similar results. **c**,**d**, Shannon indexes (**c**) and relative abundance (**d**) of endophytic bacterial microbiota at the phylum level and at class level for Proteobacteria of in Col-0, *tip1* and *snc1* leaves based on 16S rRNA gene amplicon sequence profiling. *n* = 11 (Col-0), *n* = 20 (*tip1*) and *n* = 17 (*snc1*) biological replicates for analysis of leaf endophytic bacterial microbiota. The centre lines of the box plot represent means, the box edges are the 75th and 25th percentiles, whiskers extend to 10–90 percentiles, and dots are outliers; one-way ANOVA with Tukey’s HSD test. **e**,**f**, Normalized expression levels of *PR1* (**e**) and *FRK1* (**f**) genes in 2.5-week-old plate-grown Col-0, *tip1* and *snc1* plants. *PP2AA3* expression was used for normalization. Results represent the mean values ± s.e.m. of four biological replicates. Each biological replicate is a pool of two seedlings. Statistical analysis by one-way ANOVA with Tukey’s HSD test. Experiment was independently performed twice with similar results. Source data

**Extended Data Fig. 5:**
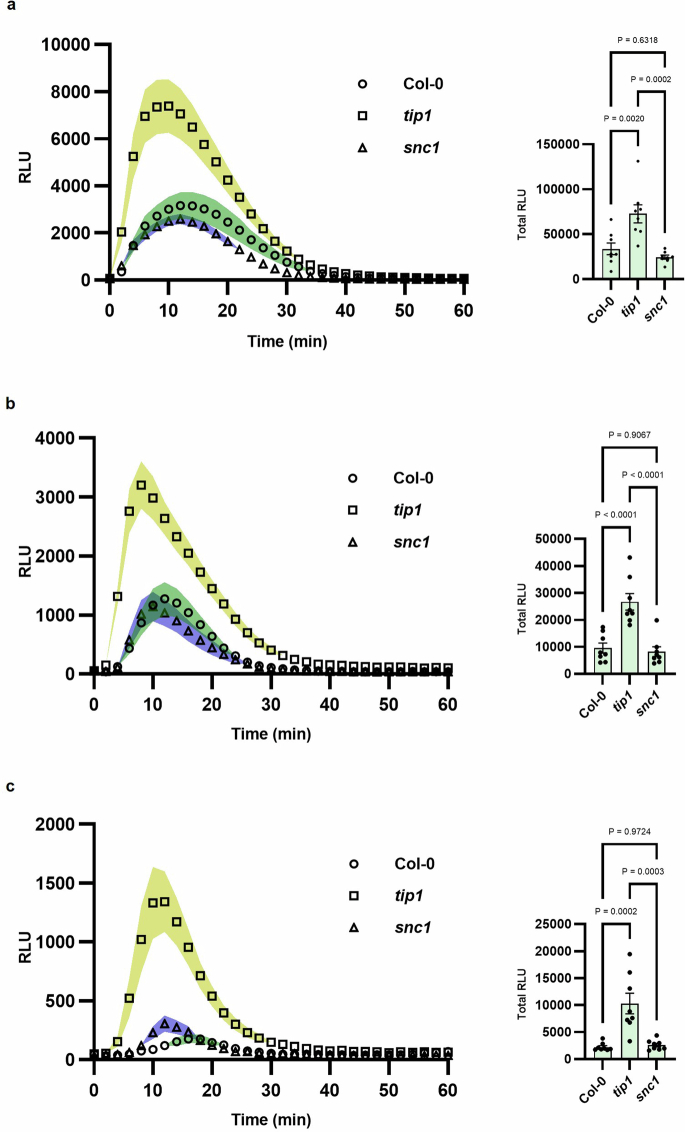
ROS burst dynamics induced by PTI elicitors. ROS burst dynamics induced by 100 nM of flg22 (**a**), elf18 (**b**) and AtPep1 (**c**) in 4-week-old soil grown plants. Results represent the mean values ± SEM (n = 8 plants). Bar plots show cumulative ROS production over 60 min (right). Results represent the mean values ± SEM of eight biological replicates. Statistical analysis was done by one-way ANOVA with Tukey’s HSD test. Experiment was independently performed twice times with similar results. Source data

As expected, *snc1* mutant plants had a dwarf morphology (Fig. 4a, top panel) and constitutively elevated immune-related marker gene expression (Fig. 3a,b). Both *snc1* and *tip1* showed enhanced resistance to the virulent pathogen *Pst* DC3000 (Fig. 3c). In addition, *tip1* leaves showed significantly higher levels of PTI-associated ROS production, as induced by flg22, elf18^33^ (an EF-Tu-derived peptide) and AtPep1^34^ (an endogenous damage-associated peptide) compared with *snc1* and wild-type Col-0 leaves (Extended Data Fig. 5). However, *snc1* and *tip1* mutant plants behaved differently in response to non-pathogenic bacteria. As shown in Fig. 3d, *tip1* single mutant plants were more susceptible to the non-pathogenic *Pst* Δ*hrcC* mutant strain, whereas *snc1* plants were marginally more resistant to the *Pst* Δ*hrcC* mutant compared with wild-type Col-0 plants.

**Extended Data Fig. 6:**
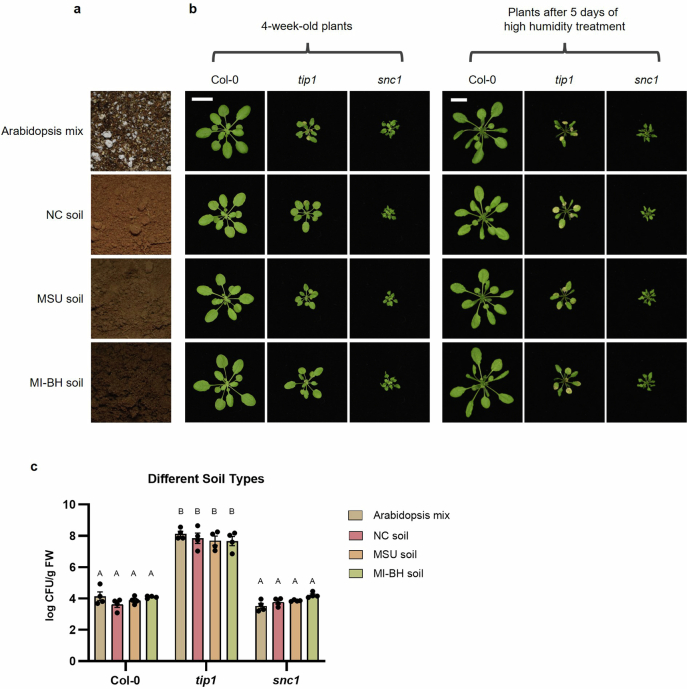
Effects of different soil types on *tip1* and *snc1* plants. **a**, soil appearance of Arabidopsis mix, North Carolina (NC) soil, Michigan State University (MSU) soil, and Michigan Benton Harbor (MI-BH) soil. **b**, Images of four-week-old plants growing on pots prepared using indicated soil types (left) and plant images after five days high humidity condition ( ~ 95% RH; right). **c**, Population sizes of endophytic leaf microbiota after five days high humidity condition ( ~ 95% RH). Results represent the mean values ± SEM (n = 4 biological replicates; each biological replicate contains leaves from one plant). Different letters represent a significant difference (p < 0.05, two-way ANOVA with Tukey’s HSD test). Exact p-values for all comparisons are shown in the Source Data. Experiment was independently performed twice with similar results. Source data

Furthermore, while most *tip1* leaves showed severe chlorotic lesions when shifted to high-humidity condition for 5 days, *snc1* mutant plants were morphologically similar under either ambient or high-humidity conditions (Fig. 4a). In addition, enumeration of leaf endophytic microbiota showed that *snc1* plants carried similar levels of culturable bacteria inside their leaves as that of Col-0 plants, whereas *tip1* plants had more than 1,000-fold increase of endophytic bacterial load compared with Col-0 after higher humidity shift (Fig. 4b). In addition to *Arabidopsis* potting soil mix, different leaf endophytic microbiota levels between *tip1* and *snc1* mutants were observed across diverse soil types from Michigan and North Carolina (Extended Data Fig. 6), suggesting that the dysbiotic phenotypes associated with the *tip1* mutant plants were robust and irrespective of soil sources and/or the microbiota associated with these soils. Along with variances in the microbiota level, the leaf microbiota compositions in *tip1* and *snc1* plants were also different. Profiling bacterial communities inside Col-0, *tip1* and *snc1* leaves using 16S ribosomal RNA gene amplicon sequencing revealed that, compared with Col-0, *tip1* plants had substantially reduced leaf endophytic microbiota diversity (Fig. 4c) with overwhelming high relative abundance of Gammaproteobacteria (Fig. 4d and Supplementary Data 2). By contrast, *snc1* plants had a diverse leaf endophytic microbiota composition, similar to that of Col-0 plants (Figs. 4c,d). Finally, when grown in aseptic agar plates, *snc1* plants continued to show heightened immune-related marker gene expression in the absence of microbes, whereas *PR1* and *FRK1* expression in *tip1* mutant plants greatly subsided to close to the low levels observed in wild-type Col-0 plants (Figs. 4e,f).

**Extended Data Fig. 7:**
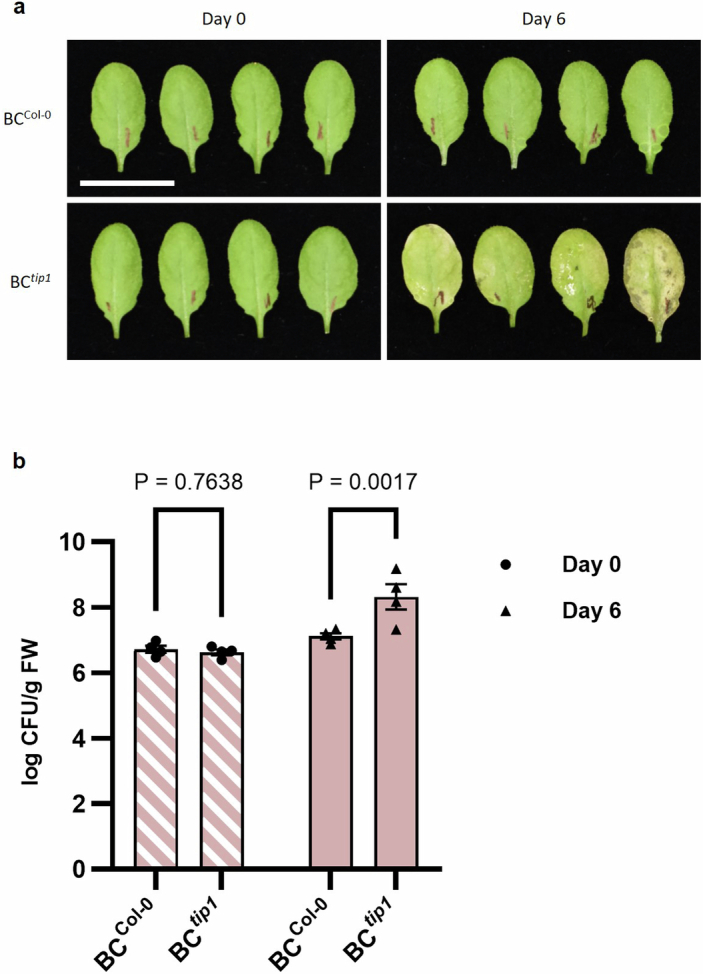
Effects of bacterial bulk culture (BC) collected from dysbiotic *tip1* plants on wild type plants. **a**, Col-0 leaf images on Day 0 and Day 6 after infiltrated with BC^Col-0^ or BC^*tip1*^. **b**, Population sizes of endophytic leaf microbiota immediate (Day 0; left) and six days (Day 6; right) after BC infiltrations. Results represent the mean values ± SEM (n = 4 biological replicates; each biological replicate contains 1-2 leaves from one plant). Statistical analysis was done by two-way ANOVA with Fisher’s LSD test. Experiment was independently performed twice with similar results. Source data

Next, we retrieved bulk culturable endophytic bacteria from *tip1* leaves following a previous study^35^. These *tip1*-associated endophytic leaf bacteria, in bulk, when infiltrated into leaves caused disease-like chlorosis in wild-type Col-0 plants (Extended Data Fig. 7), suggesting that microbiota assembled in the *tip1* mutant can cause dysbiotic plant phenotypes.

### Role of microbiota in *Arabidopsis* lesion-mimic autoimmunity

**Fig. 5:**
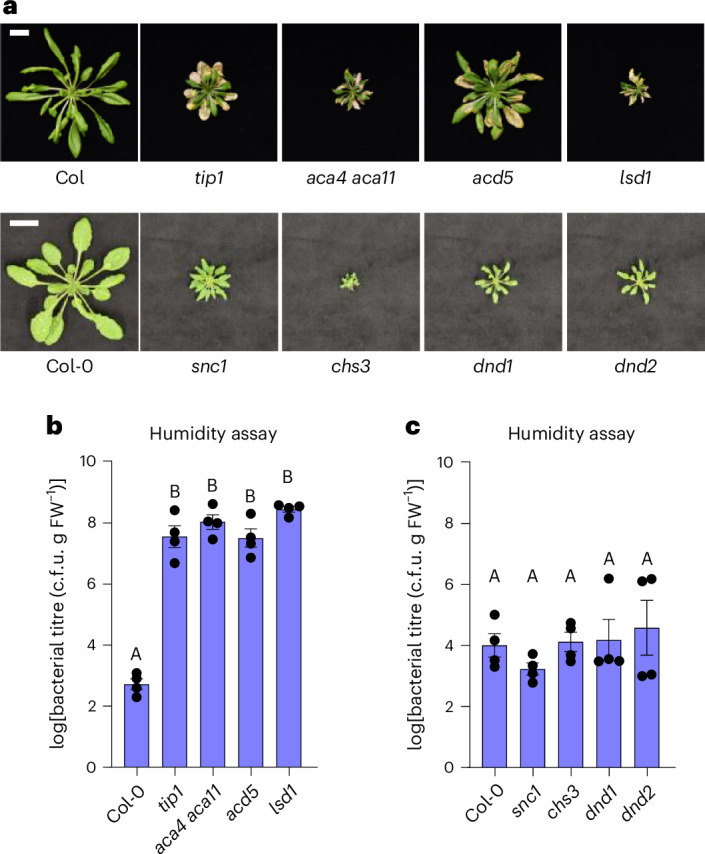
The appearance and leaf microbiota phenotypes of *Arabidopsis* autoimmune mutants. **a**, Images of 4-week-old, soil-grown *Arabidopsis* autoimmune mutants exposed to high humidity (~95% RH) for 5 days. Top panel: Col-0, *tip1* and three previously identified ‘lesion-mimic’ autoimmune mutants; bottom panel: Col-0, *snc1* and three previously identified autoimmune mutants that showed no visible lesions. Scale bar, 2 cm. **b**, Population sizes of endophytic leaf microbiota after 5 days of plant growth under high-humidity condition (~95% RH) in *tip1* and three previously identified ‘lesion-mimic’ autoimmune mutants. **c**, Population sizes of endophytic leaf microbiota after 5 days of plant growth under high humidity in *snc1* and three previously identified autoimmune mutants with no visible lesions. Results represent the mean values ± s.e.m. of four biological replicates; each biological replicate contains one to three leaves from one plant. Different letters represent a significant difference (*P* < 0.05, one-way ANOVA with Tukey’s HSD test). Exact *P* values for all comparisons are shown in the Source data. Experiment was independently performed twice with similar results. Source data

**Extended Data Fig. 8:**
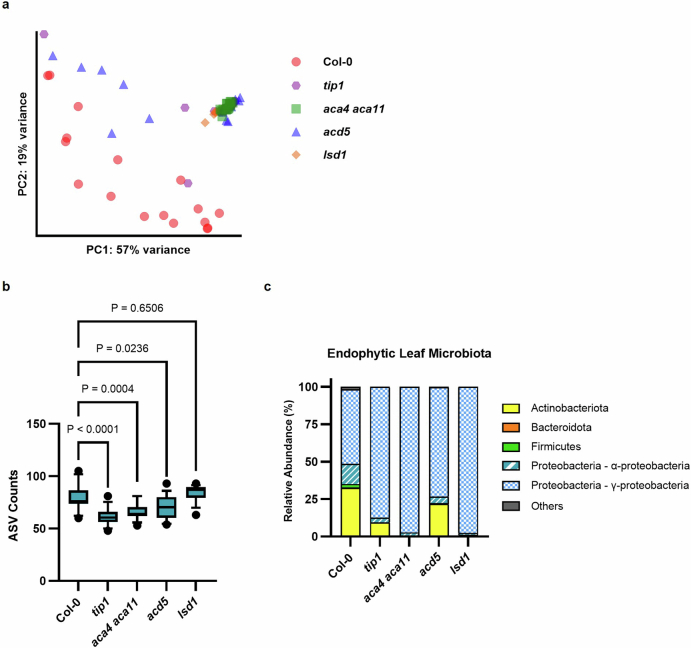
Endophytic bacterial microbiota profiling in *tip1* and *tip1*-like autoimmune mutants. **a**, PCoA of weighted UniFrac distances obtained from 16 S rRNA gene sequence profiles of endophytic bacterial microbiota in Col-0, *tip1* and *tip1*-like autoimmune mutants under high humidity for 5 days. Pairwise PERMANOVA results are provided in the Source Data. **b**, The number of ASVs indicating endophytic bacterial microbiota alpha diversity in the indicated genotypes. n = 17 (Col-0) and n = 16 (for *tip1* and *tip1*-like mutant plants). The center lines of the box plot represent means, the box edges are the 75^th^ and 25^th^ percentiles, whiskers extend to 10-90 percentiles, and dots are outliers. Statistical analysis was done by one-way ANOVA with Dunnett’s test. **c**, Relative abundance of bacterial populations at the phylum level. Members of Proteobacteria phylum are further separated into class. Source data

The interesting contrast in microbiota dependency for autoimmune phenotypes between the *tip1* and *snc1* mutants prompted us to further investigate whether there is a broad connection between microbiota dependency and autoimmunity in other reported *Arabidopsis* autoimmune mutants^36,37^ (see Supplementary Table 3 for list of mutants and their stock numbers). Based on whether they possessed tissue lesions when grown in potting soil under standard growth chamber conditions, we found that the *tip1*-like autoimmune mutant category includes (1) the *aca4 aca11* double mutant, which harbours mutations on two vacuolar calcium ion pumps ACA4 and ACA11 (ref. ^38^), (2) the *acd5* mutant, which carries mutation in a ceramide kinase^39,40^ and (3) the *lsd1* mutant, which has a defective zinc-finger protein in *Arabidopsis*^41^. The *snc1*-like autoimmune mutant category consists of (1) the *chs3* mutant, which carries a gain-of-function mutation in a Toll/interleukin-1 receptor (TIR)-type NLR immune receptor^42^, (2) *dnd1* (ref. ^43^) and (3) *dnd2* (ref. ^44^) mutants, which carry mutations in two cyclic nucleotide-gated cation channels. Mutant plants in the *tip1*-like category showed severe leaf lesions (Fig. 5a, top panel) and harboured high levels of endophytic leaf microbiota under high humidity (Fig. 5b). Principal coordinates analysis of weighted UniFrac distances from 16S rRNA gene sequencing of endophytic leaf microbiota revealed that bacterial communities associated with the *tip1*-like mutants mostly clustered together and apart from those associated with wild-type Col-0 plants (Extended Data Fig. 8a). In addition, bacterial communities associated with most *tip1*-like mutants showed a significant reduction in alpha diversity, as evidenced by lower richness compared with Col-0 (Extended Data Fig. 8b). Albeit to different degrees, mutant plants of the *tip1*-like category carried higher levels of Gammaproteobacteria, most notably two genera, *Pseudomonas* and *Stenotrophomonas*, than Col-0 plants (Extended Data Fig. 8c and Supplementary Data 3). Mutant plants in the *snc1*-like category had no visible lesions (Fig. 5a, bottom panel) and carried low levels of endophytic leaf microbiota similar to wild-type Col-0 plants (Fig. 5c).

It is worth noting that leaf endophytic microbiota profiling showed that *tip1* mutant plant samples collected at Michigan State University (MSU) carried one dominant ASV (an unspecified species in *Pseudomonas* sp; Supplementary Data 2), whereas *tip1* plant samples collected at Duke University showed three dominant ASVs (*Pseudomonas nitroreducens*, an unspecified species in *Pseudomonas* and an unspecified species *Stenotrophomonas*; Supplementary Data 3). We observed dysbiosis at both locations, suggesting that the dysbiotic phenotypes in *tip1* mutant plants are robust, albeit with somewhat different enriched ASVs.

**Fig. 6:**
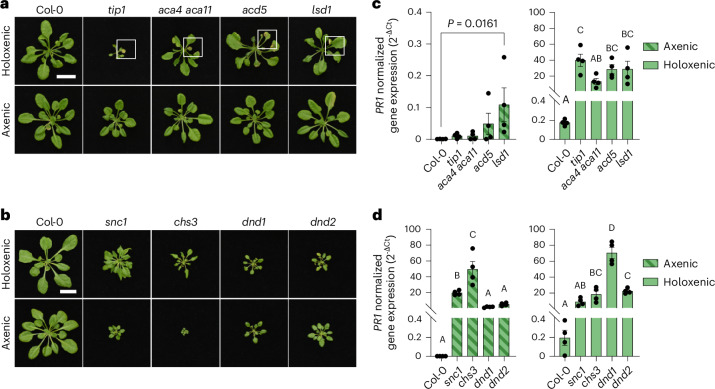
Microbiota dependency for autoimmunity in *Arabidopsis* autoimmune mutants. **a**, Five-week-old Col-0, *tip1* and three previously identified lesion-mimic autoimmune mutants grown in GnotoPots under holoxenic (top panel) or axenic (bottom panel) conditions. Scale bar, 2 cm. Zoomed-in images (white squares) on leaf lesions are shown in Supplementary Figure 2a. **b**, Five-week-old Col-0, *snc1* and three previously identified autoimmune mutants that showed no visible lesions were grown in GnotoPots under holoxenic (top panel) or axenic (bottom panel) conditions. Scale bar, 2 cm. **c**,**d**, *PR1* expression in *tip1* and three previously identified lesion-mimic autoimmune mutants (**c**) and *snc1* and three autoimmune mutants (**d**) grown in GnotoPots for 5 weeks under axenic (left; with diagonal stripe pattern) or holoxenic (right) conditions. Results represent the mean values ± s.e.m. of four biological replicates. Each biological replicate is a pool of two plants. Different letters represent a significant difference. Statistical analysis was performed using one-way ANOVA with Fisher’s least significant difference (LSD) test. Exact *P* values for all comparisons are shown in the Source data. Experiment was independently performed twice with similar results. Source data

To further characterize a possible microbiota dependency of autoimmune phenotypes in these two categories of mutants, we grew mutant plants in the absence (axenic) or presence (holoxenic) of a natural ‘MSU’ soil-derived microbiota using GnotoPots, a peat-based gnotobiotic system as described^26^. Growing under the holoxenic condition, lesion-mimic mutants showed various degrees of chlorosis and lesions (Fig. 6a, top panel, and Supplementary Fig. 2a) and heightened immune-related marker gene expression (Fig. 6c, right panel). However, in the absence of microbiota, these lesion-mimic autoimmune mutants showed neither chlorosis nor lesions (Fig. 6a, bottom panel), and their immune marker gene expression also subsided to a low basal level, with the exception of *lsd1* (Fig. 6c, left panel). By contrast, mutants in the *snc1*-like category have high basal *PR1* expression even in the axenic condition. For example, the *chs3* mutant showed heightened *PR1* expression regardless of the presence or absence of microbiota (Fig. 6d), behaving similarly to the *snc1* mutant, which shows microbiota-independent autoimmunity. Furthermore, compared with microbiota-induced lesions and immune-related gene expression in lesion mimic mutants, the autoimmune dwarf phenotype of *chs3* mutants was noticeably alleviated in the presence of microbiota (Fig. 6b), again similar to the *snc1* mutant. In *dnd1* and *dnd2* mutants, *PR1* expression was elevated to a higher level when they were grown in the presence of microbiota compared with when grown in the axenic condition (Fig. 6d), behaving intermediately between lesion mimic type and *snc1* type. Together, these results suggest that there are at least two types of autoimmunity in plants: one depends on microbiota for autoimmune phenotypes, as exemplified by the *tip1* mutant, and the other is independent of microbiota, as exemplified by the *snc1* mutant. *dnd1* and *dnd2* mutants share with *snc1*-type in that they do not show lesions in the presence of microbiota and have a high basal defence gene expression in the absence of microbiota, although defence gene expression is further enhanced in the presence of microbiota.

**Extended Data Fig. 9:**
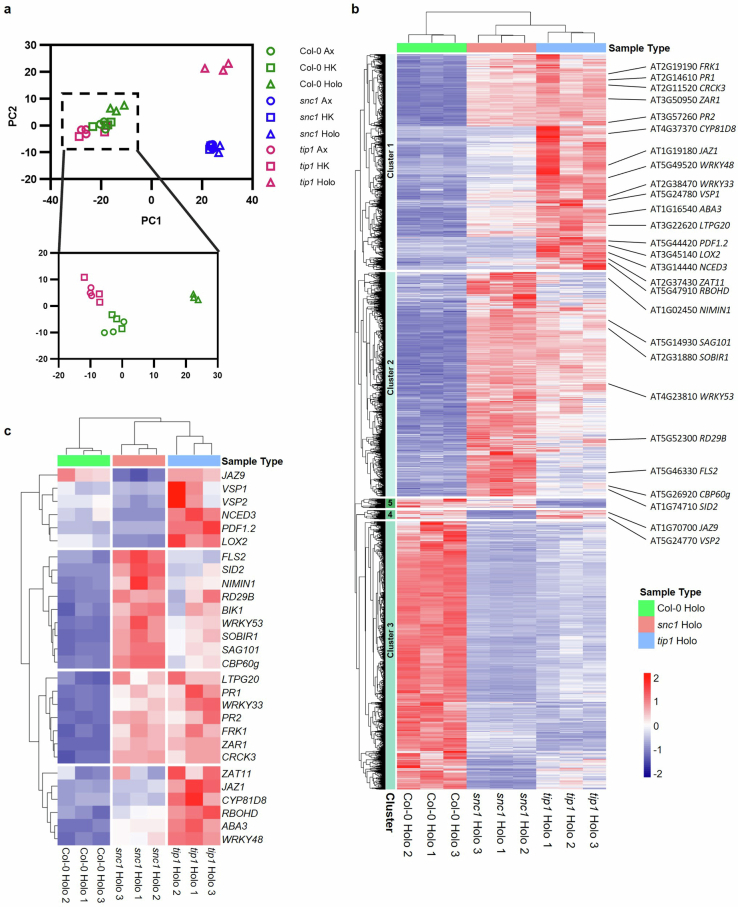
Transcriptomic analysis of Col-0, *tip1* and *snc1* plants grown in GnotoPots. **a**, PCA analysis of genes expressed in Col-0, *tip1* and *snc1* plants grown in GnotoPots under axenic (Ax), heat-killed “MSU” microbial community (HK) or live “MSU” microbial community (holoxenic; Holo) conditions. Zoomed-in image contains PCA analysis of Col-0 under all three conditions and *tip1* grown in GnotoPots under Ax and HK conditions. **b**, Heat map of DEGs significantly changed between Col-0 and *tip1* and/or between Col-0 and *snc1* plants grown in GnotoPots under “MSU” microbial community holoxenic condition. Clusters 1 and 2 are enriched for defence-related Gene Ontology (GO) terms, whereas cluster 3 is enriched for growth and photosynthesis-related GO terms. See Supplementary Data 4 for the complete list of genes and their normalized counts. **c**, Selected defence-related gene expression in Col-0, *tip1* and *snc1* plants grown in GnotoPots under “MSU” microbial community holoxenic condition. See Supplementary Table 5 for the gene IDs.

To capture genome-wide gene expression in *tip1* and *snc1* plants beyond the *PR1* and *FRK1* marker genes and to further characterize gene expression patterns associated with the distinct microbiota dependency of autoimmune phenotypes in these two mutants, we conducted transcriptomic profiling of these two mutants grown in the absence (axenic) or presence (holoxenic) of a natural ‘MSU’ soil-derived microbiota in GnotoPots. Heat-killed ‘MSU’ soil-derived microbial communities were included to investigate whether autoimmune phenotypes could be activated by microbe-associated molecular patterns associated with heat-killed microbes. Principal component analysis of gene expression data revealed distinct expression patterns in Col-0, *tip1* and *snc1* plants (Extended Data Fig. 9a). The respective gene expression patterns in Col-0 and *tip1* plants under axenic and heat-killed conditions clustered together, suggesting that in the absence of live microbes, *tip1* mutant plants behaved similarly as wild-type Col-0 and that heat-killed microbes were not sufficient to alter gene expression patterns in Col-0 and *tip1* plants (Extended Data Fig. 9a). Under the holoxenic condition, gene expression pattern in *tip1* plants is distinct from that under the axenic condition or Col-0 plants under holoxenic condition, which is consistent with the dramatic microbiota-dependent autoimmunity phenotype. By contrast, gene expression patterns in *snc1* plants under all three growth conditions clustered closely and away from Col-0 and *tip1* gene expression patterns, which is consistent with the microbiota-independent autoimmune phenotype in the *snc1* mutant.

Furthermore, in accordance with increased *PR1* and *FRK1* marker gene expression (Figs. 3a,b), PTI/salicylic acid-associated immune genes were induced, to various degrees, in *tip1* as well as *snc1* plants under the holoxenic condition (Extended Data Fig. 9b). It is worth noting that jasmonate-associated genes appear to be uniquely enriched in *tip1* plants but not in *snc1* plants under the same condition probably related to tissue damage in the *tip1* mutant (Extended Data Fig. 9c).

### Role of microbiota in autoimmunity in wild accessions

**Fig. 7:**
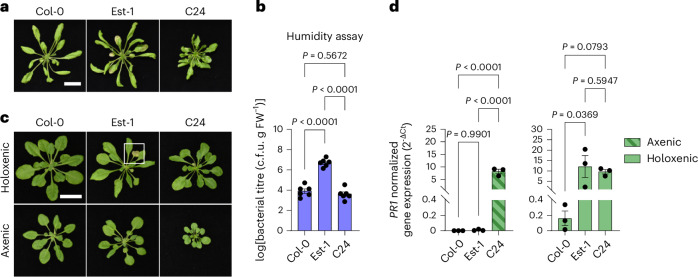
Microbiota dependency for autoimmunity in *A. thaliana* natural accessions. **a**, Images of 5-week-old, potting soil-grown *A. thaliana* accessions (Col-0, Est-1 and C24) exposed to high humidity (~95% RH) for 7 days. Scale bar, 2 cm. **b**, Population sizes of leaf endophytic microbiota after 7 days of plant growth under high-humidity condition (~95% RH). Results represent the mean values ± s.e.m. of six plants. Statistical analysis was performed using one-way ANOVA with Tukey’s HSD test. Experiment was independently performed twice with similar results. **c**, Five-week-old *Arabidopsis* accessions grown using GnotoPots under holoxenic (top panel) or axenic (bottom panel) conditions. Scale bar, 2 cm. Zoomed-in image (white square) on Est-1 leaf lesions is shown in Supplementary Figure 2b. **d**, *PR1* expression in *Arabidopsis* accessions grown in GnotoPots under axenic (left; with diagonal stripe pattern) or holoxenic (right) conditions. Results represent the mean values ± s.e.m. of three biological replicates. Each biological replicate is a pool of two plants. Statistical analysis was performed using one-way ANOVA with Fisher’s LSD test. Experiment was independently performed twice with similar results. Source data

Autoimmunity has been observed in natural *Arabidopsis* populations/accessions^45^. For example, *Arabidopsis thaliana* accessions Est-1 and C24 have constitutively elevated defence gene expression and enhanced disease resistance toward the virulent pathogen *Pst* DC3000 when grown in potting soil^46,47^. We were therefore interested in knowing whether autoimmune phenotypes of natural accessions are dependent on microbiota. When grown in potting soil under ambient humidity, Est-1 showed chlorosis and lesions on older leaves, whereas C24 had curly leaves and small stature but did not show chlorosis or lesions (Supplementary Fig. 3). However, like the *tip1* mutant, Est-1 leaves showed stronger leaf lesions under high humidity (Fig. 7a) and harboured a higher level of endophytic bacterial microbiota compared with Col-0 plants (Fig. 7b). By contrast, C24 plants did not show chlorosis nor necrosis under ambient (Supplementary Fig. 3) or high humidity (Fig. 7a); in addition, similarly to the *snc1* mutant, C24 plants maintained similar levels of endophytic leaf bacterial microbiota compared with Col-0 (Fig. 7b). The phenotypic resemblance between *tip1* and Est-1 and between *snc1* and C24 prompted us to investigate whether microbiota is required for the autoimmune phenotypes in Est-1 and C24. As shown in Fig. 7c, under holoxenic condition, Est-1 shows lesions on leaves, albeit to a lesser extent compared with Est-1 grown under the conventional potting soil growth condition (Supplementary Fig. 3 and Supplementary Fig. 2b). However, like *tip1*, Est-1 plants did not show leaf lesions under the axenic condition. Furthermore, like the *tip1* mutant, the heightened *PR1* expression in Est-1 subsided to a low level when grown in the axenic condition (Fig. 7d, left plot). Conversely, like the *snc1* mutant, C24 plants had elevated *PR1* expression regardless of growth in the presence or absence of a microbial community (Fig. 7d). Another similarity between the *snc1* mutant and C24, which is in contrast to the *tip1* mutant, is the alleviation of their stunted growth morphology in the presence of microbiota (Figs. 6b and 7c).

## Discussion

A healthy microbiome can play a vital role in initiating, training and maintaining host immune homeostasis. In return, the host immune system can fine tune its immune strength to accommodate commensal/symbiotic microbes and to prepare for a robust immune response against pathogenic microbe invasion. As plants spend most of their life interacting with a vast number of commensal microbes and occasionally encountering pathogens, understanding the intricate interplays between plant immunity and the endophytic commensal microbiota is important for explaining how plants dial their plant immune system to maximize the effectiveness of plant immune responses to nurture beneficial microbes and/or fight against pathogens. In this study, we conducted a forward genetic screen aimed at identifying *Arabidopsis* mutants that cannot maintain a normal leaf microbiota. Among putative mutants isolated was *grm1*, which we characterized in detail.

The *grm1* mutant contains a missense mutation in the *TIP1* gene that encodes S-acyltransferase. The first mutant allele of *TIP1* was isolated in a genetic screen for mutants that had defects in root hair development^48^. The causal mutation was later mapped to *At5g20350* (ref. ^49^). *At5g20350*/*TIP1* encodes one of the 24 DHHC (Asp-His-His-Cys)-containing S-acyltransferases and it is also one of the two ankyrin repeats containing DHHC S-acyltransferases in *Arabidopsis*. Ankyrin repeats containing DHHC S-acyltransferases are conserved across eukaryotes (Supplementary Fig. 4 and Supplementary Table 4). Both the *Arabidopsis* TIP1 protein and the human HIP14 (huntingtin-interacting protein 14; zinc finger DHHC-type palmitoyltransferase 17 (zDHHC17)) have been shown to be functional orthologues of yeast Akr1p^49,50^. Akr1p and zDHHC17 are involved in vesicle trafficking. For example, acylation of yeast Yck2 protein by Akr1p is required for proper localization of Yck2 to the plasma membrane via secretory vesicles, and Yck2p’s membrane association is essential for its biological function in yeast morphogenesis^51^.

Before this study, however, the connection between TIP1 and leaf microbiota homeostasis was not known. A comprehensive *Arabidopsis* acylome using multiple tissue types identified close to 1,100 putative S-acylated proteins^52^. Thirty-seven per cent of identified proteins overlapped with those identified in a previous study which used *Arabidopsis* root cell culture^53^. Notably, many of the identified proteins have been demonstrated to be associated with microbe perception and plant immune responses^53^, including FLS2 and EFR receptors^54–56^. While abolishing predicted acylation sites on these receptor-like kinases attenuated their immune responses, it is not known whether they are substrates of TIP1. Our data showed that ROS response activated by flg22, elf18 and AtPep1, which are perceived FLS2 (ref. ^57^), EFR^58^ and AtPEPR1 (ref. ^34^), respectively, were not compromised in *tip1* plants (Extended Data Fig. 5). This suggested that FLS2, EFR and AtPEPR1 are not likely the direct substrates of TIP1 S-acyltransferase. It is worth noting that *TIP1* gene expression itself can be induced by flg22 regardless of the presence of the microbiota (Extended Data Fig. 4). In light of our findings on a genetic connection between *TIP1* and maintenance of a normal leaf microbiota, future research is needed to identify specific TIP1 substrate(s) that is required for microbiota homeostasis in *Arabidopsis* leaves.

A key finding in this study is that the *tip1* mutants not only are unable to control the proliferation or maintain a normal composition of a leaf microbiota but also show dysbiosis-associated tissue damages and autoimmunity in the presence of microbiota (Fig. 4). The microbiota-dependent autoimmune phenotypes of the *tip1* mutant led us to broadly examine a potential connection between microbiota and previously reported ‘autoimmune’ mutants in *Arabidopsis*. Based on how they respond to the existence of microbiota, it appears that autoimmune mutants in *Arabidopsis* can be divided into at least two classes (Supplementary Fig. 5). One class, exemplified by the *tip1* mutant, shows microbiome-dependent autoimmunity in leaves. The autoimmune phenotypes in this class largely disappeared when grown in the axenic conditions. Given that these mutants also have an increased level and altered composition of endophytic leaf microbiota, this result suggests that the autoimmune phenotypes in this class of mutants are a consequence of harbouring an overabundant and/or aberrant microbial community. The other class of autoimmunity in our study is independent of microbiota and is represented by the *snc1* mutant. The autoimmune phenotypes of this class do not require the presence of microbiota. That is, they have small statures and high *PR1* expression regardless of the presence or absence of microbial communities. It is worth noting that the presence of microbiota alleviates the stunted growth morphology of *snc1* and *chs3* (Fig. 6b), which is in striking contrast to those of the *tip1* class (Fig. 6a). *TIP1* and other autoimmune-causing genes are themselves differentially expressed in Col-0 leaves under axenic, heat-killed, holoxenic conditions, albeit with no consistent patterns (Supplementary Fig. 6).

While the autoimmune phenotypes of *snc1* and *chs3* mutants are independent of microbiota and their stunted growth morphology can be partially alleviated by the presence of microbiota, not all NLR autoimmune mutants may behave in a similar fashion. For example, in *Arabidopsis* No-0 ecotype, which carries mutations in *SLH1* (S*ensitive to Low Humidity 1*), a TIR-type NLR with a WRKY domain at its C-terminus showed normal growth on agar plate but showed growth arrest and development of necrotic lesions when transferred to a microbe-rich soil environment^59^. However, in this case, it remains to be determined whether the autoimmune phenotype differences of No-0 plants between growth on agar plate and growth in soil is indeed caused by microbiota. In addition, observations of autoimmune phenotypes attributed to NLR proteins were made in hybrid necrosis^60^, a term to describe the phenomenon of incompatibility in the F1 progeny from interspecific or intraspecific crosses. In at least one case, this hybrid necrosis/hybrid autoimmunity caused by *NLR* genes is independent of microbiota^61^. Finally, a recent study in root-associated microbiota showed that mutations in the *Arabidopsis PSKR1* (*Phytosulfokin Receptor 1*) gene led to autoimmunity. It is worth noting that the autoimmunity phenotype in this case is associated with reduced Pseudomonads in the rhizosphere^62^. It will be of interest in the future to determine whether there are microbiota changes in the leaves of the *pskr1* mutant.

We find it interesting that microbiota-dependent and -independent autoimmunity can also be observed in *Arabidopsis* natural accessions. Although autoimmunity is often associated with fitness trade-offs at the individual plant level^10,11^, the presence of heightened basal immunity in natural accessions suggests a fitness advantage at the population level^47^. For example, if a devastating disease spreads through a largely susceptible *Arabidopsis* population, accessions such as C24 and Est-1 may be able to survive and reproduce to avoid extinction of the entire population. The microbiota-dependent and -independent expression of autoimmunity in Est-1 versus C24, as observed in our study (Fig. 7), may reflect different paths by which the two types of autoimmunity have convergently evolved in natural populations under different abiotic and biotic pressures. Future research is needed to determine whether the TIP1-dependent pathway plays a role in natural accessions that show microbiota-dependent autoimmunity.

Overall, results from this study begin to illustrate conceptual parallels in microbiota interactions with plants and animals and may have broad implications in understanding host–microbiota interactions in general. In mammalian–microbiota interactions, for example, dysbiotic shifting/reducing diversity in microbiome composition is often associated with inflammation and dysregulated immune responses that fail to distinguish self from non-self, which are characteristics of autoimmune disorders^63–65^. The similarities in autoimmune symptoms between the *Arabidopsis tip1* mutant and mammalian inflammatory autoimmunity are notable that they include a dysbiotic microbial community, tissue lesions and dysregulated immune responses. The most renowned substrate of zDHHC17, the human homologue of *Arabidopsis* TIP1, is huntingtin^50,66^. In light of the connection between the *tip1* mutation and autoimmunity in *Arabidopsis*, it would be interesting for future research to examine whether mutations in zDHHC17 or other TIP1 orthologues are associated with dysbiosis and/or whether dysbiosis is involved in Huntington’s disease development. Indeed, recent reports have suggested an association between Huntington’s disease and dysbiosis^67^. The advance in biochemistry studies of zDHHC17 (refs. ^68–70^) and the more easily amenable mutant studies at the whole organismal level in *Arabidopsis* should facilitate further understanding of a possibly broad role of DHHC S-acyltransferases in microbiome homeostasis and immunity across the kingdoms of life.

## Methods

### Plant materials and growth conditions

All seeds were surface-sterilized using 15% diluted bleach (containing final concentration of 1.2% active ingredient sodium hypochlorite (NaOCl)) before being sown onto potting soil. All plants were grown under a 12 h day/12 h night regimen with 100 µE light intensity and ~50% RH, unless otherwise indicated. See Supplementary Table 3 for a complete list of *Arabidopsis* mutants and accessions used in this study.

For plant images and microbiota quantification on plants grown on different soil types, soil was sourced from Miscanthus plot at MSU (42° 43′ 01.2″ N, 84° 27′ 45.8″ W)^26^, Michigan Benton Harbor (42° 05′ 06.2″ N, 86° 21′ 12.1″ W) and North Carolina (36° 13′ 07.2″ N, 79° 10′ 45.5″ W). ‘*Arabidopsis* mix’ potting soil was prepared in-house by mixing equal volumes of SureMix (Michigan Grower Products), perlite and vermiculite. After preparing pots using different soil types, all pots were treated with NEMAforce SF for insect pest control before sowing surface-sterilized seeds.

For experiments using GnotoPots, a peat-based gnotobiotic system^26^, nutrients were supplemented with buffered half-strength Linsmaier and Skoog liquid media (0.5× LS, pH 5.7; Caisson Labs LSP03). Soil for natural microbiota inoculation was collected from MSU. Holoxenic plants were inoculated with soil slurry (10 g soil per litre of 0.5× LS), whereas axenic plants were inoculated with 0.5× LS liquid medium. Heat-killed natural microbiota input was prepared by inoculating with an autoclaved soil slurry.

### Genetic screen

Roughly 30,000 *Arabidopsis bak1-5 bkk1-1 cerk1-2* (*bbc*) seeds were mutagenized using 0.2% ethyl methanesulfonate (EMS). Mutagenized M1 seeds were sown on soil and allowed to grow to set seeds. Seeds from two to three M1 plants were pooled, and approximately 1,700 pools were collected. This EMS population was estimated to cover the *Arabidopsis* genome more than 10 times. The primary screen was conducted by seedling flood-inoculation assay^71^. In brief, roughly 50 M2 seeds from each pool were sown onto 0.5× LS agar plates followed by flooding 3-week-old seedlings with 1 × 10^8^ c.f.u. ml^−1^ *Pst* D28E (suspended in 2.5 mM MgCl_2_ and 0.015% Silwet L-77) for 4 min. After 4 min, inoculum was removed, and plates were returned to Percival chambers for disease symptom development. Mutants showing signs of chlorosis and/or necrosis were transplanted to potting soil and transferred to a growth chamber to collect M3 seeds. Secondary screen was conducted by monitoring symptoms after either syringe infiltration of non-pathogenic *Pst* Δ*hrcC* mutant strain to 4-week-old soil-grown M3 plants or after growth in holoxenic condition in the FlowPot gnotobiotic plant growth system^26^.

### Mutation identification using mapping-by-sequencing approach

To identify the causative mutation in the *grm1* mutant, a mapping-by-sequencing population was generated by backcrossing the *grm1* mutant with the *bbc* mutant. All four F1 plants showed *bbc*-like morphology, suggesting that the mutant trait is recessive. F1 plants were selfed to produce F2 populations. Of 674 F2 plants screened, 633 had *bbc*-like morphology, and 41 were *grm1*-like. This ratio deviates from the 3:1 single nuclear gene inheritance pattern. However, mapping-by-sequencing data does not support the idea that the mutant phenotype in *grm1* plants was caused by two or more unlinked loci as the allele frequency only peaks at around 7 Mb on chromosome 5 (Extended Data Fig. 2a); the G-to-A mutation in the *TIP1* (*At5g20350*) gene is tightly associated with the *grm1* phenotype (Extended Data Fig. 2 and Supplementary Table 1). A literature search found that a similar genetic inheritance pattern deviation was observed in the characterization of the first mutant allele of *TIP1* (*tip1-1*; ref. ^48^). The authors suggested that ‘deficiency in the number of homozygous *tip1* mutant seeds’ as a potential cause of such inheritance ratio deviation.

### Genetic complementation of the *grm1* mutant

High-fidelity PCR was performed using Col-0 genomic DNA as template and with a sense primer covering roughly 2 kb upstream of the *At5g20350* start codon and an anti-sense primer roughly 1 kb downstream of the stop codon. Cloned PCR product was inserted into pDONR207 entry vector and verified using Sanger sequencing. Recombination reaction was conducted using verified entry clone and pMDC123 destination vector to create the *TIP1* genomic clone driven by the *TIP1* native promoter. The construct was transformed into the *grm1* mutant plants via floral dipping method^72^ using *Agrobacterium tumefaciens* GV3101 as the vehicle strain. See Supplementary Table 2 for the primer sequences used in this study.

### Quantification of endophytic leaf bacterial microbiota

Four-week-old potting soil-grown plants were sprayed with distilled water and fully covered with a clear dome to maintain high humidity (~95% RH) for 5 days (or 7 days for natural *Arabidopsis* accessions). After high humidity treatment, one to three leaves from each plant were surface sterilized with diluted bleach (containing final concentration of 0.3% active ingredient NaOCl) for 1 min followed by two rinses with sterile water. Surface-sterilized leaves were blotted dry using paper towels and weighted. Sterile water was added to leaf samples, which were homogenized using TissueLyser II (QIAGEN) for 2 × 45 s at 30 Hz. Homogenized samples were serial diluted and spotted onto R-2A plates (Sigma-Aldrich catalogue number 17209) supplemented with cycloheximide (15 mg l^−1^) and 0.5% methanol to enumerate culturable colonies.

The *tip1* mutant plants showed most prominent dysbiosis phenotypes under high humidity; we therefore performed most of our dysbiosis-related experiments under high humidity.

### Preparation of bacterial bulk culture

Lysates prepared from surface-sterilized leaf tissues (10 µl autoclaved Milli-Q water per mg of leaf fresh weight) from Col-0 and *tip1* plants treated with high humidity (~95% RH) were mixed with glycerol to final concentration of 20%, aliquoted and stored in −80 °C.

Seven days before the experiment, one tube of Col-0 and one tube of *tip1* leaf tissue lysate glycerol stock were taken out from the −80 °C, serial diluted and plated onto R-2A plates (supplemented with 15 mg l^−1^ cycloheximide and 0.5% methanol). Plates were incubated at 10 °C. On the day of the experiment, plates containing mostly isolated colonies were used to prepare bacterial bulk culture (BC) as described^35^ with modifications. To prepare bacterial BC, bacteria were scraped from the agar with a sterile loop and transferred to 15 ml tubes containing 2.5 mM MgCl_2_; cells were pelleted down at 5,000 × *g* for 5 min and resuspended in 1 ml 2.5 mM MgCl_2_. BC^Col-0^ was diluted to optical density at 600 nm of 0.05 and BC^*tip1*^ to 0.04, which gave ~5 × 10^7^ c.f.u. ml^−1^.

Four-week-old soil-grown Col-0 plants were hand-infiltrated using a needleless syringe with either BC^Col-0^ or BC^*tip1*^. After infiltration, day 0 samples were collected, and the remaining infiltrated plants were returned to growth chambers and kept at high humidity (~95% RH) for symptom progression. Samples were collected 6 days post inoculation (d.p.i.).

### 16S rRNA gene amplicon sequencing

For this experiment, all consumables and kits came from the same lot to avoid any background contamination or variations. Seeds of indicated *Arabidopsis* genotypes were surface sterilized using 15% bleach (1.2% active ingredient NaOCl) and washed twice using autoclaved Milli-Q water before sowing onto potting soil. Plants were grown in growth chambers. Four-week-old plants were sprayed with distilled water and kept at ≥95% RH for 5 days. One to two leaves per plant were collected and surface-sterilized using diluted bleach (0.3% active ingredient NaOCl) for 1 min, followed by two washes using autoclaved Milli-Q water. Excess water on leaf surfaces was blotted dry, put into Safe-Lock Eppendorf tubes containing 3 mm zirconium beads (Glen Mills), snap froze in liquid nitrogen and stored at −80 °C.

Total DNA (host and microbes) was extracted using DNeasy PowerSoil Pro Kit (QIAGEN catalogue number 47014) following the manufacturer’s protocol. Extracted DNA was used as template for PCR amplification of the v5/v6 region of 16S rRNA gene using 799 F and 1,193 R primers (see Supplementary Table 2 for the sequence of primers) and high-fidelity AccuPrime Taq DNA Polymerase (Invitrogen catalogue number 12346086). Amplified products were run in 1% agarose gels to separate bacterial and chloroplast 16S rRNA gene amplicons (~400 bp) from the mitochondrial 18S amplicon (~750 bp). DNA in the ~400 bp band was recovered using the Zymoclean Gel DNA Recovery Kit (Zymo Research D4008). Concentration of recovered DNA was measured using the Quant-iT PicoGreen dsDNA Assay Kits (Invitrogen P7589) and normalized to 3–8 ng µl^−1^ for sample submission. Library preparation and sequencing using MiSeq (Figs. 1 and 4) or NovaSeq (Extended Data Fig. 8) platforms (2 × 250 bp paired-end format) was conducted by the Genomic Core Facility at MSU.

### 16S rRNA gene amplicon sequencing data processing

Raw Illumina data for 16S rRNA gene amplicon sequencing were processed as described previously^20^ using QIIME2 version 2022.2^73^. In brief, primer sequences were removed using Cutadapt^74^ followed by filtering, denoising and creating an ASV table by DADA2 (ref. ^75^). For data from NovaSeq platform, ASVs contributing to less than 0.005% of the total or only present in a single sample were removed from analysis. Taxonomic assignment of each ASV was performed using a Naive Bayes classifier^76^, pre-trained on the SILVA 16S rRNA gene reference database^77,78^ (release 138; https://www.arb-silva.de/) formatted for QIIME using RESCRIPt^79^. Unassigned sequences and sequences annotated as mitochondria and chloroplast were removed. Diversity analyses were performed within QIIME2. Samples were rarified to 1,952 and 74,693 reads for data from MiSeq and NovaSeq platforms, respectively, for calculating diversity metrics. The entire sequence analysis workflow is available on GitHub (https://github.com/BradCP/Roles-of-microbiota-in-autoimmunity-in-Arabidopsis).

### Bacterial infection assays

Four-week-old soil-grown plants were hand-infiltrated using a needleless syringe with either *Pst* DC3000 at 1 to 2 × 10^5^ c.f.u. ml^−1^ or *Pst* Δ*hrcC* at 2 to 3 × 10^6^ c.f.u. ml^−1^. After infiltration, excess bacterial suspensions were blotted dry, and plants were returned to growth chambers and kept at high humidity (~95% RH) for disease progression. Samples were collected 3 d.p.i. for *Pst* DC3000 or 5 d.p.i. for *Pst* Δ*hrcC*. To determine bacterial population in leaves, leaf discs were collected and ground in autoclaved Milli-Q water using a TissueLyser II (QIAGEN; 45 s at 30 Hz). Serial dilutions of the ground tissue lysates were spotted onto low-salt Luria–Bertani plates (10 g l^−1^ tryptone, 5 g l^−1^ yeast extract and 5 g l^−1^ NaCl; pH 7.0) with appropriate antibiotics. Colony-forming units per cm^2^ were determined for each sample.

### Reverse transcription quantitative PCR analysis gene expression

For gene expression analysis, plant tissues at the indicated conditions were collected, snap frozen in liquid N_2_ and stored at −80°C until further processing.

Total RNA was extracted from plant tissues using TRIzol Reagent (Thermo Fisher catalogue number 15596026) according to the manufacturer’s instructions. Complementary DNA synthesis was accomplished in 10 µl volume with SuperScript IV VILO Master Mix (Thermo Fisher catalogue number 11756050) according to the manufacturer’s instructions with 1 µg total RNA as input. Upon reverse transcription, the product was diluted fivefold using TE buffer (10 mM Tris–HCl pH 8.0, 1 mM EDTA). Quantitative PCR was performed in a 10 µl reaction volume containing 5 µl SYBR Green PCR master mix (Thermo Fisher catalogue number 4309155), 500 nM of each primer, and 1 µl of template cDNA using a QuantStudio 3 real-time PCR system (Applied Biosystems). *PP2AA3* was used for normalization. The primer sets used to quantify gene expression in this study are listed in Supplementary Table 2.

### Transcriptome analysis

Total RNA was extracted from plant tissues using TRI Reagent (Sigma T9424) and cleaned up using Zymo RNA Clean & Concentrator-5 (R1013). RNA Integrity was assessed using Bioanalyzer Plant RNA Pico Assay (Agilent RNA 6000 Pico Kit 5067-1513) with RIN values 7.6 ± 0.3.

Library preparation and transcriptome sequencing was complete by the MSU Genomics Core Facility. In brief, libraries were prepared from plant total RNA using the Illumina Stranded mRNA Prep, Ligation kit with IDT for Illumina RNA UD Indexes, following manufacturer’s recommendations except that half volume reactions were performed. Completed libraries were quality controlled and quantified using a combination of Qubit dsDNA HS and Agilent 4200 TapeStation HS DNA1000 assays. The libraries were pooled in equimolar amounts and the pool quantified using the Invitrogen Collibri Quantification qPCR kit. The pool was loaded onto one lane of an Illumina S4 flow cell, and sequencing was performed in a 2 × 150 bp paired-end format using a NovaSeq v1.5, 300 cycle reagent kit.

Base calling was done by Illumina Real Time Analysis v3.4.4, and output of Real Time Analysis was demultiplexed and converted to FastQ format with Illumina Bcl2fastq v2.20.0.

Abundance of transcripts was quantified via Salmon v1.2.1 (ref. ^80^), and differential expression analysis and plotting was accomplished via R packages, including DESeq2 v1.42.0, apeglm v1.24.0, ggplot2 v3.5.0 and pheatmap v1.0.12.

### Phylogenetic analysis

To find all possible TIP1 orthologues, DHHC domain and ankyrin-repeat domain of TIP1 were blasted against 131 proteomes (23 species outside of the plant kingdom and 108 species inside the plant kingdom; Supplementary Table 4), via Phytozome^81^, Ensemble^82^ and PLAZA^83^ databases. Proteins were confirmed via InterPro^84^ for the presence of both DHHC and ankyrin-repeat domains. In total, 329 proteins from 123 species, including TIP1 (AT5G20350) and TIP1 paralog (AT2G14255), were then used to construct the phylogenetic tree via maximum likelihood algorithm using MEGA7 (ref. ^85^).

### ROS burst assay

Leaf discs (4 mm in diameter) were taken from the centre of leaves from 4-week-old soil-grown plants and floated with abaxial side down in wells of a white 96-well plate containing 200 µl sterile water in each well. Plates were covered with foil, and leaf discs were kept in sterile water overnight to attenuate wounding response. After 24 h in the dark, water was removed from wells and replaced with 100 µl of an immune-eliciting solution containing 68 µg ml^−1^ luminol (Sigma, A8511), 20 µg ml^−1^ horseradish peroxidase (Sigma, P6782) and 100 nM of the indicated Pathogen/Damage-Associated Molecular Patterns (PAMP/DAMP). Luminescence measurements were collected (total photon counting) over 60 min immediately after the addition of immune-eliciting solution using a SpectraMax L microplate reader with SoftMax Pro v.7.0.3 (Molecular Devices). Total ROS was calculated for each sample in Prism v.10.0.0 (GraphPad) using the ‘area under curve’ analysis.

### Reporting summary

Further information on research design is available in the Nature Portfolio Reporting Summary linked to this article.

## Supplementary information

Supplementary Information Supplementary Figs. 1–6.

Reporting Summary

Supplementary Data 1 Four tables; ASV counts from 16S amplicon profiling of leaf endophytic microbiota (Tables 1–3) and differentially expressed genes of transcriptomic data (Table 4).

Supplementary Data 2 Nine tables; statistical source data for Supplementary Fig. 6.

Supplementary Table 1 Five tables; list of primers, mutant lines and gene/protein IDs as supporting information.

## Source data

Source Data Fig. 1 Statistical source data.

Source Data Fig. 2 Statistical source data.

Source Data Fig. 3 Statistical source data.

Source Data Fig. 4 Statistical source data.

Source Data Fig. 5 Statistical source data.

Source Data Fig. 6 Statistical source data.

Source Data Fig. 7 Statistical source data.

Source Data Extended Data Fig. 1 Unprocessed gel image.

Source Data Extended Data Fig. 1 Statistical source data.

Source Data Extended Data Fig. 2 Statistical source data.

Source Data Extended Data Fig. 3 Statistical source data.

Source Data Extended Data Fig. 4 Statistical source data.

Source Data Extended Data Fig. 5 Statistical source data.

Source Data Extended Data Fig. 6 Statistical source data.

## Data Availability

Raw Illumina data for 16S rRNA gene amplicon sequences for the *grm1* mutant and related controls are available in the Sequence Read Archive database (SRA) under BioProject PRJNA934331, with accession numbers SAMN33271678 to SAMN33271728. Raw Illumina data for 16S rRNA gene amplicon sequences for Col-0, *tip1* and *snc1* are available in the SRA database under BioProject PRJNA934350, with accession numbers SAMN33272493 to SAMN33272548. Raw Illumina data for 16S rRNA gene amplicon sequences for the *tip1*-like lesion-mimic autoimmune mutants are available in the SRA database under BioProject PRJNA1101553, with accession numbers SAMN40996484 to SAMN40996564. RNA-sequencing reads data have been deposited in the SRA database under BioProject PRJNA1103072, with accession numbers SAMN41039378 to SAMN41039404. Source data are provided with this paper.

## Code availability

There is no custom code generated for this study.
