# Supplementary material for: Roles of microbiota in autoimmunity in Arabidopsis leaves
Source: Nat Plants. 2024 Sep 6;10(9):1363–76. doi: 10.1038/s41477-024-01779-9 (PMC11410663; doi:10.1038/s41477-024-01779-9)
Supplement: Supplementary file 1 — Supplementary Figs. 1–6. [file 41477_2024_1779_MOESM1_ESM.pdf]

nature plants

Supplementary information https://doi.org/10.1038/s41477-024-01779-9

Roles of microbiota in autoimmunity in *Arabidopsis* leaves

In the format provided by the authors and unedited

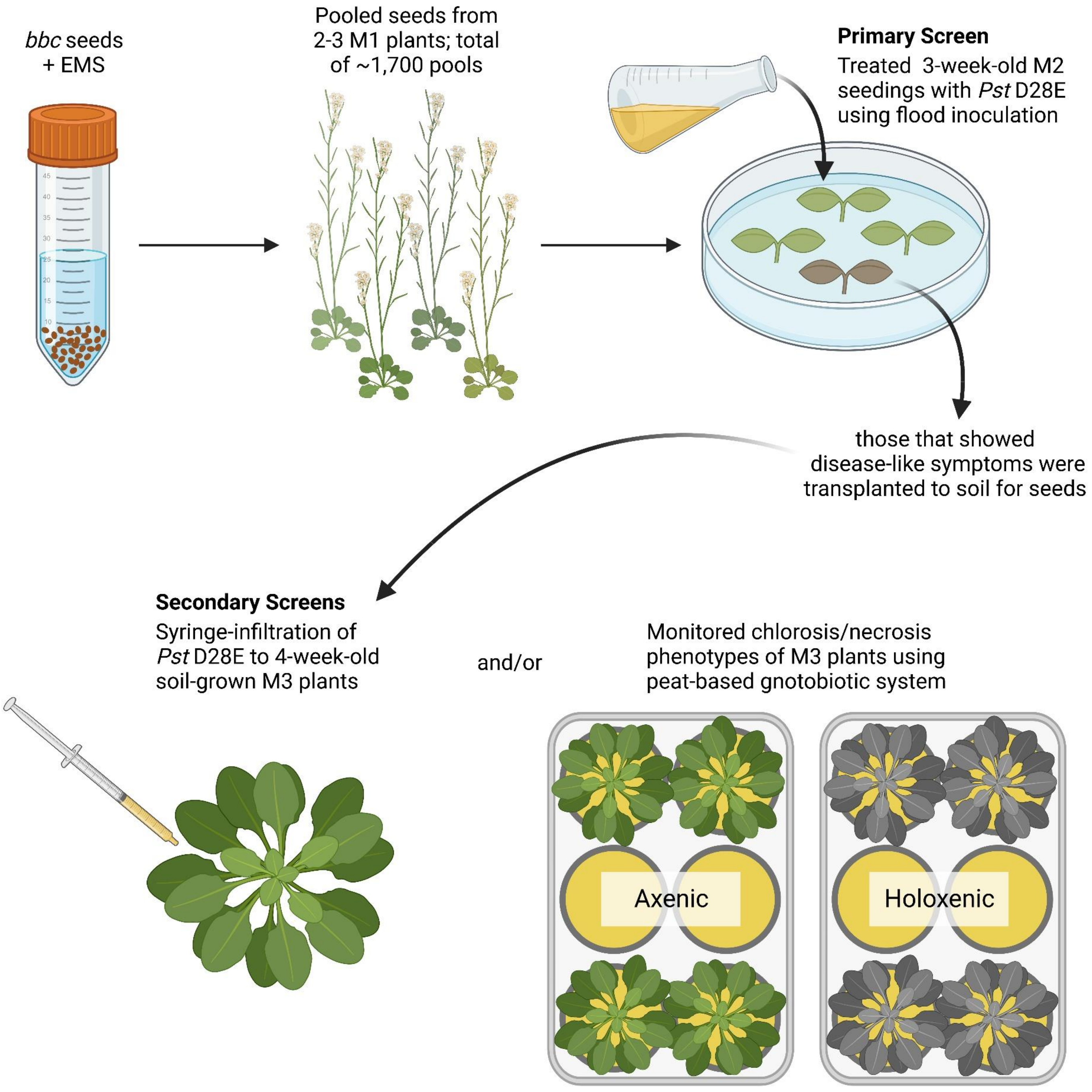

Supplementary Figure 1. A schematic diagram of the genetic screen workflow.

See Methods for detailed description of the genetic screen. Image was created with BioRender.com.

a

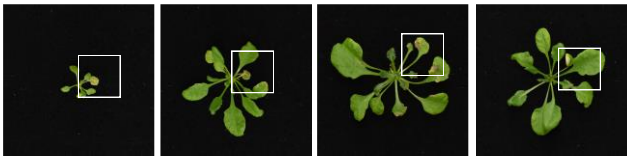

tip1 *aca4 aca11* *acd5* *lsd1*

Zoomed-in images of leaf lesions of indicated genotypes

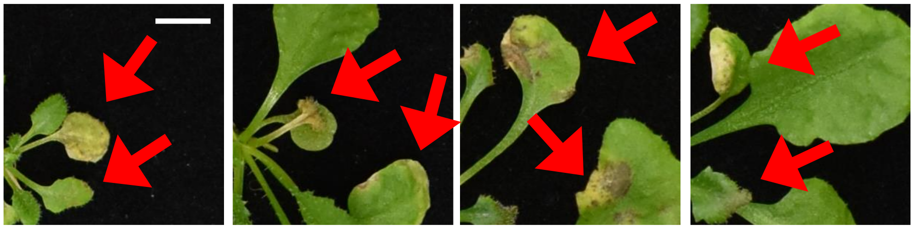

tip1 *aca4 aca11* *acd5* *lsd1*

b

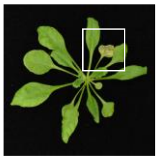

Est-1

Zoomed-in image of leaf lesions of indicated accession

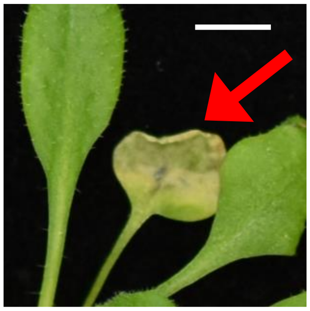

Est-1

Supplementary Figure 2. Zoomed-in images of leaf lesions on plants grown in holoxenic conditions.

a, Zoomed-in images of indicated genotypes grown on GnotoPots under holoxenic conditions. Scale bar equals 0.5 cm. **b**, Zoomed-in image of *A. thaliana* accession Est-1 grown on GnotoPots under holoxenic conditions. Scale bar equals 0.5 cm.

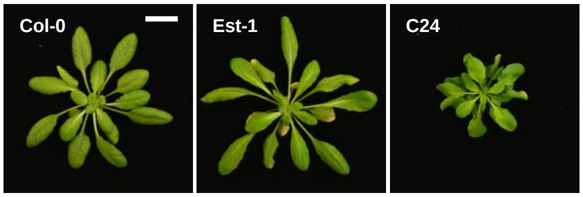

Supplementary Figure 3. Appearance of 5-week-old, soil-grown Col-0, Est-1 and C24 plants.

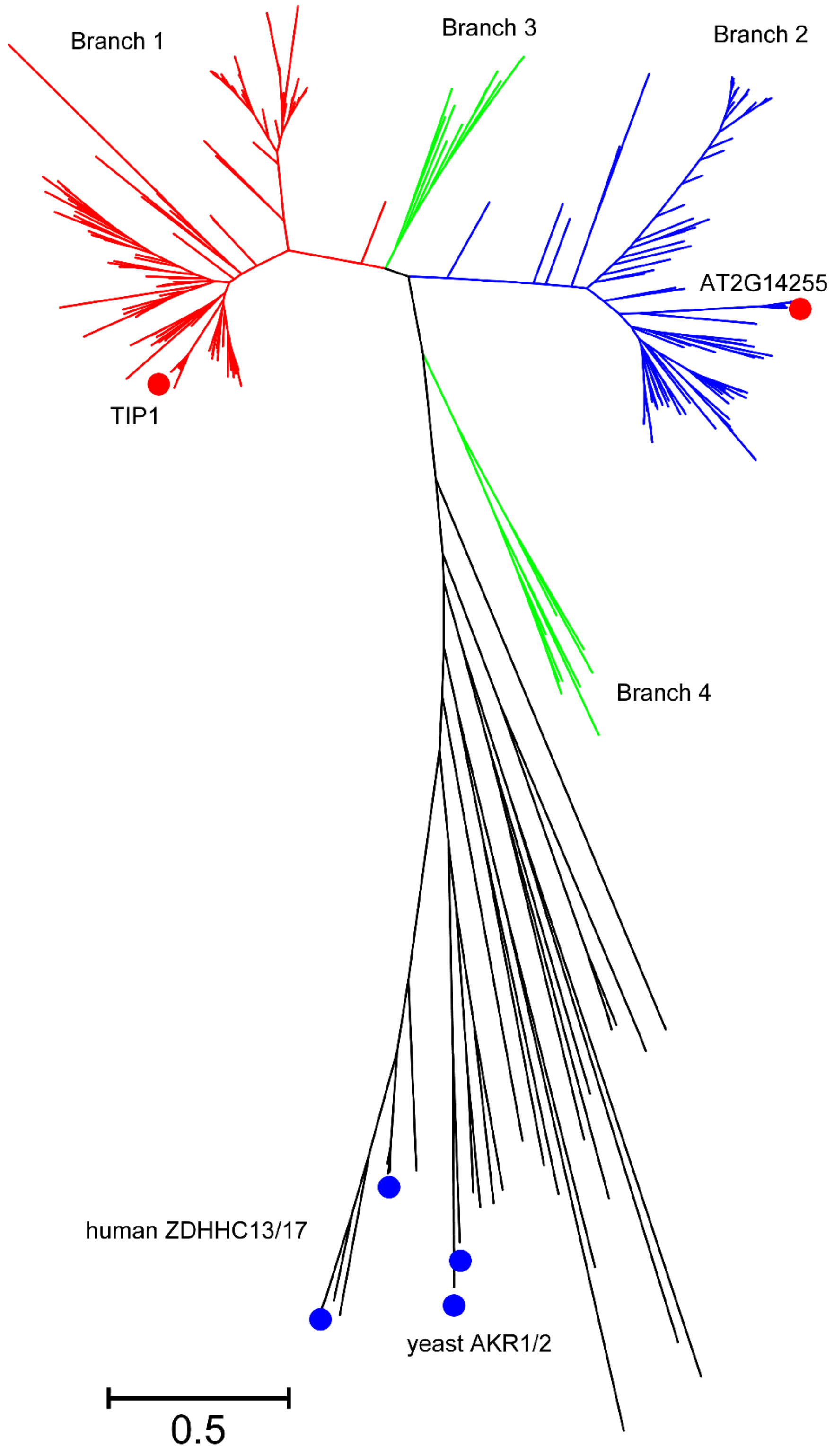

Supplementary Figure 4. Phylogenetic tree of TIP1 orthologs.

329 TIP1 orthologs from 123 species with both DHHC and ankyrin-repeat domains were selected to construct the phylogenetic tree. Arabidopsis TIP1 protein (AT5G20350) and TIP1 paralog (AT2G14255) were highlighted using red dots. Human and yeast TIP1 homologs ZDHHC13, ZDHHC17, AKR1 and AKR2 were highlighted using blue dots. Proteins within the plant kingdom could be divided into four branches, Branch 1-4. Among them, Branch 1 (red) includes proteins closer to TIP1 and Branch 2 (blue) includes proteins closer to AT2G14255. TIP1 homologous proteins were evolved as early as in chlorophyte (green) and protists (black) and developed independently within the plant kingdom (Branch 1-4) and the animal kingdom (black). The scale bar represents 0.5 substitutions per amino acid site. Protein information was listed in Supplementary Table 4.

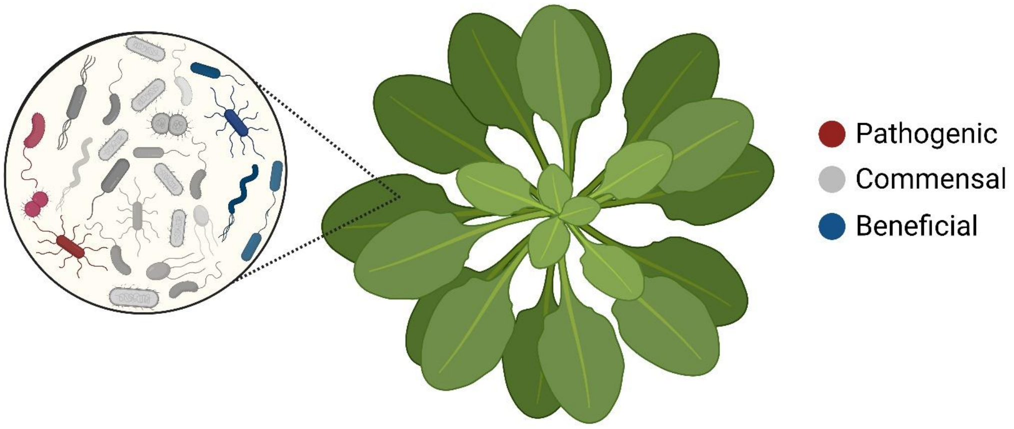

Microbiota-Independent Autoimmunity (e.g. *snc1*)

- resistant to pathogenic bacteria *Pst* DC3000
- have curled leaves and small stature
- no apparent lesions
- wild-type level of endophytic leaf microbiota
- high defence marker gene expression regardless of the presence or absence of microbiota

Microbiota-Dependent Autoimmunity (e.g. *tip1*)

- resistant to pathogenic bacteria *Pst* DC3000
- have small stature and usually associated with various severity of lesions
- substantially higher level of endophytic leaf microbiota
- tissue lesions disappeared in the absence of microbial community
- high defence marker gene expression only in the presence microbiota

Supplementary Figure 5. Characteristics of two types of autoimmunity in plants based on their microbiota dependency.

Image was created with BioRender.com

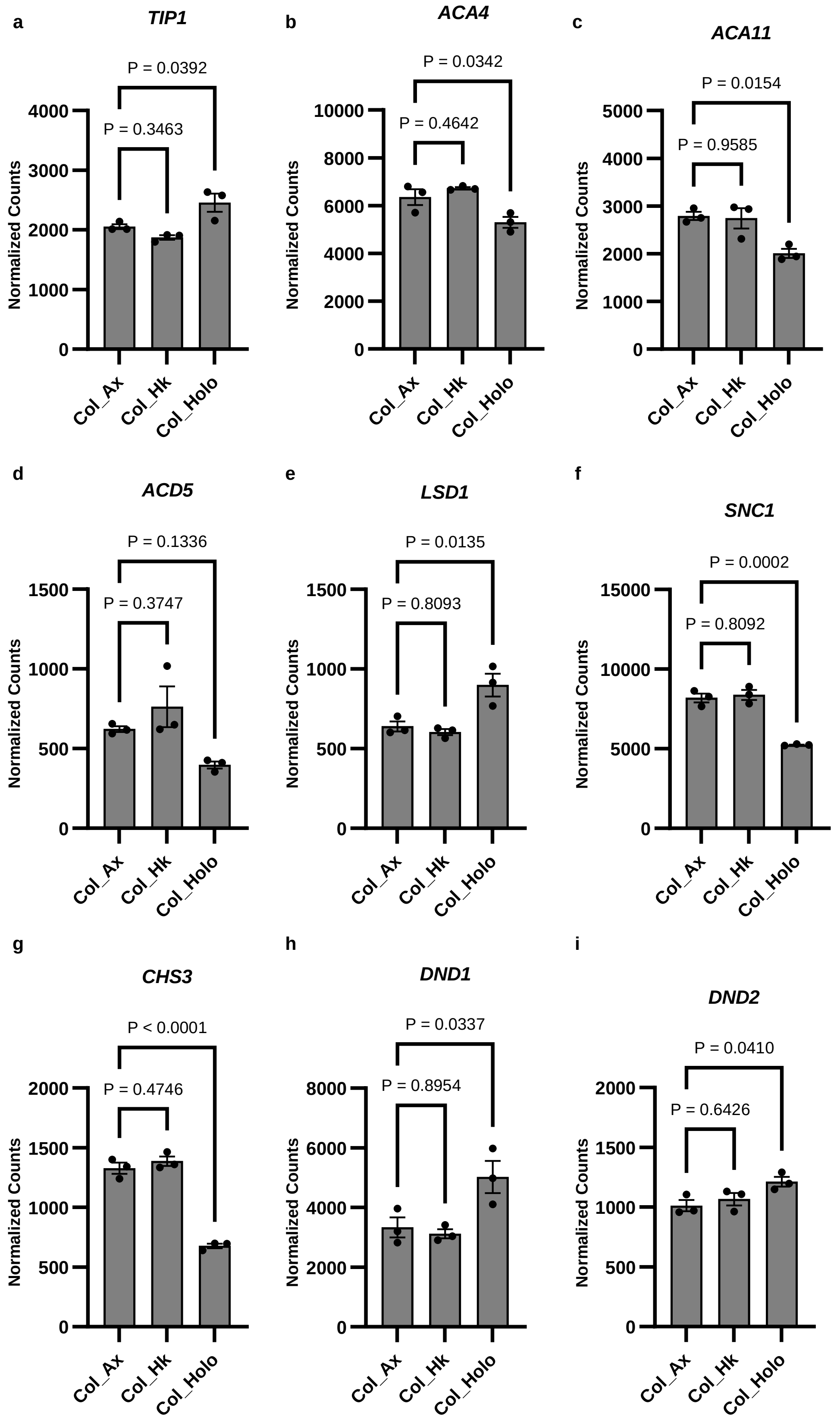

Supplementary Figure 6. *TIP1* and other autoimmune-causing gene expression in Col-0 leaves under axenic, heat-killed, and holoxenic conditions.

Gene expression pattern of *TIP1* (a), *ACA4* (b), *ACA11* (c), *ACD5* (d), *LSD1* (e), *SNC1* (f), *CHS3* (g), *DND1* (h), and *DND2* (i) in Col-0 plants under axenic (Ax), heat-killed (Hk), and holoxenic (Holo) conditions. Bar plots were generated using normalized read counts from the transcriptomic profiling data on Col-0 plants under axenic, heat-killed, and holoxenic conditions. Results represent the mean values ± SEM (n=3 biological replicates). Statistical analysis was done by one-way ANOVA with Dunnett’s test.
