# Supplementary material for: Roles of microbiota in autoimmunity in Arabidopsis leaves
Source: Nat Plants. 2024 Sep 6;10(9):1363–76. doi: 10.1038/s41477-024-01779-9 (PMC11410663; doi:10.1038/s41477-024-01779-9)
Supplement: Supplementary file 2 — Reporting Summary [file 41477_2024_1779_MOESM2_ESM.pdf]

nature portfolio

Corresponding author(s): Sheng Yang He and Yu Ti Cheng

Last updated by author(s): July 08, 2024

Reporting Summary

Nature Portfolio wishes to improve the reproducibility of the work that we publish. This form provides structure for consistency and transparency in reporting. For further information on Nature Portfolio policies, see our Editorial Policies and the Editorial Policy Checklist.

Statistics

For all statistical analyses, confirm that the following items are present in the figure legend, table legend, main text, or Methods section.

n/a	Confirmed	
☐	☒	The exact sample size (*n*) for each experimental group/condition, given as a discrete number and unit of measurement
☐	☒	A statement on whether measurements were taken from distinct samples or whether the same sample was measured repeatedly
☐	☒	The statistical test(s) used AND whether they are one- or two-sided<br>*Only common tests should be described solely by name; describe more complex techniques in the Methods section.*
☒	☐	A description of all covariates tested
☐	☒	A description of any assumptions or corrections, such as tests of normality and adjustment for multiple comparisons
☐	☒	A full description of the statistical parameters including central tendency (e.g. means) or other basic estimates (e.g. regression coefficient) AND variation (e.g. standard deviation) or associated estimates of uncertainty (e.g. confidence intervals)
☐	☒	For null hypothesis testing, the test statistic (e.g. *F*, *t*, *r*) with confidence intervals, effect sizes, degrees of freedom and *P* value noted<br>*Give P values as exact values whenever suitable.*
☒	☐	For Bayesian analysis, information on the choice of priors and Markov chain Monte Carlo settings
☒	☐	For hierarchical and complex designs, identification of the appropriate level for tests and full reporting of outcomes
☒	☐	Estimates of effect sizes (e.g. Cohen's *d*, Pearson's *r*), indicating how they were calculated

Our web collection on statistics for biologists contains articles on many of the points above.

Software and code

Policy information about availability of computer code

Data collection	16S rDNA amplicon sequence: Illumina MiSeq System (Figure 1 and Figure 4); MiSeq v2 500 cycle: Illumina Real Time Analysis (RTA) v1.18.54, Illumina Bcl2fastq v2.20.0.<br>16S rDNA amplicon sequence: Illumina NovaSeqSystem (Extended Data Figure 8); NovaSeq 6000 v1.5 500 cycle: Illumina Real Time Analysis (RTA) v3.4.4, Illumina Bcl2fastq v2.20.0.<br>Gene expression: Applied Biosystems QuantStudio 3 Real-Time PCR System<br>DNA gel imaging: Invitrogen iBright 1500 system<br>ROS: SoftMax Pro v7.0.3<br>Transcriptomic analysis: Base calling was done by Illumina Real Time Analysis (RTA) v3.4.4 and output of RTA was demultiplexed and converted to FastQ format with Illumina Bcl2fastq v2.20.0.
Data analysis	Statistics and graph production: GraphPad Prism 10 software<br>Description of published software used for analyzing 16S rDNA amplicon sequence data are cited in the Method section. These include, QIIME 2 (version 2022.2), Cutadapt v4.1 and DADA2 v1.22.0.<br>Total ROS: GraphPad Prism 10 software.<br>Transcriptomic analysis: Abundance of transcripts was quantified via Salmon v1.2.1 and differential expression analysis and plotting was accomplished via R packages, including DESeq2 v1.42.0, apeglm v 1.24.0, ggplot2 v 3.5.0, and pheatmap v 1.0.12.

For manuscripts utilizing custom algorithms or software that are central to the research but not yet described in published literature, software must be made available to editors and reviewers. We strongly encourage code deposition in a community repository (e.g. GitHub). See the Nature Portfolio guidelines for submitting code & software for further information.

Data

Policy information about availability of data

All manuscripts must include a data availability statement. This statement should provide the following information, where applicable:

- Accession codes, unique identifiers, or web links for publicly available datasets
- A description of any restrictions on data availability
- For clinical datasets or third party data, please ensure that the statement adheres to our policy

The source data needed to evaluate this paper are available in the main text and Supplementary Information. Uncropped gel image is provided in Source Data with this paper.

Raw Illumina data for 16S rDNA amplicon sequences for the grm1 mutant and related controls are available in the Sequence Read Archive database (SRA) under BioProject PRJNA934331, accession numbers SAMN33271678 to SAMN33271728.
Raw Illumina data for 16S rDNA amplicon sequences for Col-0, tip1 and snc1 samples are available in the SRA database under BioProject PRJNA934350, accession numbers SAMN33272493 to SAMN33272548.
Raw Illumina data for 16S rDNA amplicon sequences for the tip1-like lesion-mimic autoimmune mutants are available in the SRA database under BioProject PRJNA1101553, accession numbers SAMN40996484 to SAMN40996564.
RNA-sequencing reads data have been deposited in SRA database under BioProject PRJNA1103072 accession numbers SAMN41039378 to SAMN41039404.

Human research participants

Policy information about studies involving human research participants and Sex and Gender in Research.

Reporting on sex and gender	Not Applicable
Population characteristics	Not Applicable
Recruitment	Not Applicable
Ethics oversight	Not Applicable

Note that full information on the approval of the study protocol must also be provided in the manuscript.

Field-specific reporting

Please select the one below that is the best fit for your research. If you are not sure, read the appropriate sections before making your selection.

☒ Life sciences ☐ Behavioural & social sciences ☐ Ecological, evolutionary & environmental sciences

For a reference copy of the document with all sections, see nature.com/documents/nr-reporting-summary-flat.pdf

Life sciences study design

All studies must disclose on these points even when the disclosure is negative.

Sample size	Sample size and statistical analyses are described in the relevant figure legends. Sample size was determined based on experimental trials and with consideration of previous publications on similar experiments to allow for confident statistical analyses.<br>Paasch, B. C. et al. Nat Plants 9, 1468-1480 (2023).<br>Chen, T. et al. Nature 580, 653-657 (2020).<br>Xin, X. F. et al. Nature 539, 524-529 (2016).<br>There were no statistical methods used to predetermine sample sizes.
Data exclusions	No data that pass quality control were excluded from statistical analysis.
Replication	The number of independent replication for each experiment is described in the relevant figure legends. Two independent experiments were performed for all assays. Results were ensured to be reproducible in all repeats with the same trend.
Randomization	Soil-grown plants of different genotypes were grown side by side in environmentally controlled growth chambers to minimize unexpected environmental variations during growth and experimentation. Gnotobiotic plants were grown within Microboxes placed side-by-side in environmentally controlled growth chambers to minimized unexpected environmental variations. Additionally, flats and Microboxes were rotated periodically to randomize the effect of localized environmental differences within a chamber. Leaves of similar age were collected from plants at the indicated ages and assessed randomly.
Blinding	Researchers were not blinded to allocation during experiments and outcome assessment. This is in part because different plant genotypes and treatments under study exhibit very distinct phenotypes visually; blinding was not possible. Routine practices included more than

one author observing/assessing phenotypes, whenever possible.

Reporting for specific materials, systems and methods

We require information from authors about some types of materials, experimental systems and methods used in many studies. Here, indicate whether each material, system or method listed is relevant to your study. If you are not sure if a list item applies to your research, read the appropriate section before selecting a response.

Materials & experimental systems

n/a	Involved in the study
☒	☐ Antibodies
☒	☐ Eukaryotic cell lines
☒	☐ Palaeontology and archaeology
☒	☐ Animals and other organisms
☒	☐ Clinical data
☒	☐ Dual use research of concern

Methods

n/a	Involved in the study
☒	☐ ChIP-seq
☒	☐ Flow cytometry
☒	☐ MRI-based neuroimaging
