# Supplementary figures and images for: Roles of microbiota in autoimmunity in Arabidopsis leaves
Source: Nat Plants. 2024 Sep 6;10(9):1363–76. doi: 10.1038/s41477-024-01779-9 (PMC11410663; doi:10.1038/s41477-024-01779-9)

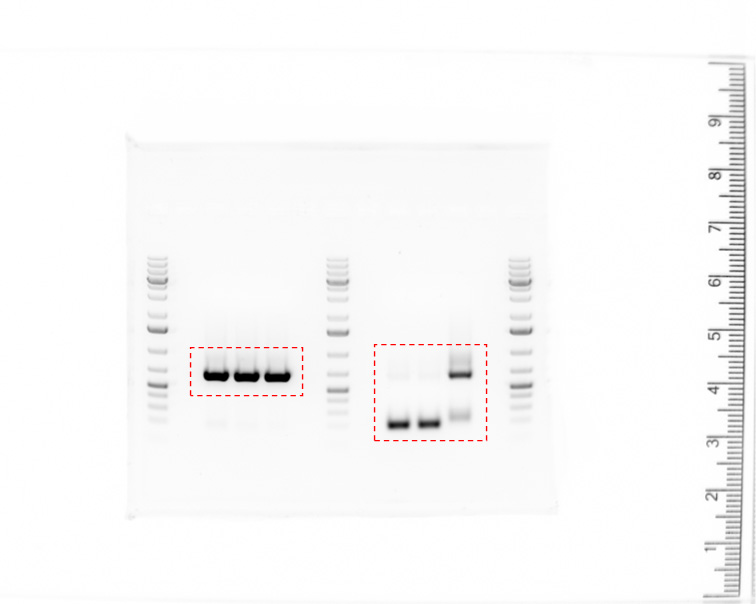

Supplement: Supplementary file 13 — Unprocessed gel image. [file 41477_2024_1779_MOESM13_ESM.jpg]
